# Single-domain antibodies neutralize ricin toxin intracellularly by blocking access to ribosomal P-stalk proteins

**DOI:** 10.1016/j.jbc.2022.101742

**Published:** 2022-02-17

**Authors:** Timothy F. Czajka, David J. Vance, Simon Davis, Michael J. Rudolph, Nicholas J. Mantis

**Affiliations:** 1 Department of Biomedical Sciences, University at Albany, Albany, New York, USA; 2 Division of Infectious Diseases, Wadsworth Center, New York State Department of Health, Albany, New York, USA; 3 New York Structural Biology Center, New York, New York, USA

**Keywords:** toxin, ribosome-inactivating protein, single-domain antibody, X-ray crystallography, phage display, AUC, area under the curve, BSA–P2C11, P2C11 peptide–bovine serum albumin BSA conjugate, CDR, complementarity-determining region, EF, elongation factor, ER, endoplasmic reticulum, HRP, horseradish peroxidase, IVT, *in vitro* translation, LNP, lipid nanoparticle, mAb, monoclonal antibody, PBST, PBS containing 0.1% Tween-20, PDB, Protein Data Bank, RIP, ribosome-inactivating protein, RTA, ricin toxin A subunit, RTB, ricin toxin B subunit, Sc, shape complementary, SPR, surface plasmon resonance, SRL, sarcin–ricin loop, TMB, 3,3′,5,5′-tetramethylbenzidine, TNA, toxin-neutralizing activity

## Abstract

During ricin intoxication in mammalian cells, ricin's enzymatic (RTA) and binding (RTB) subunits disassociate in the endoplasmic reticulum. RTA is then translocated into the cytoplasm where, by virtue of its ability to depurinate a conserved residue within the sarcin–ricin loop (SRL) of 28S rRNA, it functions as a ribosome-inactivating protein. It has been proposed that recruitment of RTA to the SRL is facilitated by ribosomal P-stalk proteins, whose C-terminal domains interact with a cavity on RTA normally masked by RTB; however, evidence that this interaction is critical for RTA activity within cells is lacking. Here, we characterized a collection of single-domain antibodies (V_H_Hs) whose epitopes overlap with the P-stalk binding pocket on RTA. The crystal structures of three such V_H_Hs (V9E1, V9F9, and V9B2) in complex with RTA revealed not only occlusion of the ribosomal P-stalk binding pocket but also structural mimicry of C-terminal domain peptides by complementarity-determining region 3. *In vitro* assays confirmed that these V_H_Hs block RTA–P-stalk peptide interactions and protect ribosomes from depurination. Moreover, when expressed as “intrabodies,” these V_H_Hs rendered cells resistant to ricin intoxication. One V_H_H (V9F6), whose epitope was structurally determined to be immediately adjacent to the P-stalk binding pocket, was unable to neutralize ricin within cells or protect ribosomes from RTA *in vitro*. These findings are consistent with the recruitment of RTA to the SRL by ribosomal P-stalk proteins as a requisite event in ricin-induced ribosome inactivation.

Ricin toxin is the archetype of the large and diverse family of medically important plant and bacterial ribosome-inactivating proteins (RIPs). A byproduct of castor beans (*Ricinus communis*), ricin toxin consists of two equal sized subunits, ricin toxin A subunit (RTA) and ricin toxin B subunit (RTB), that associate through electrostatic and covalent interactions (1, 2). RTB is a bivalent Gal/GalNAc-specific lectin that traffics RTA from the plasma membrane to the endoplasmic reticulum (ER) (3). Within the ER, the interchain disulfide bond linking RTA and RTB is reduced by protein disulfide isomerase, and RTA is then retrotranslocated in an unfolded state across the ER membrane and into the cytoplasm (4, 5, 6, 7). From that point forward, RTA functions as a RIP, triggering the ribotoxic stress response and programmed cell death pathways (8, 9, 10, 11).

The mode of action of RTA in inactivating mammalian ribosomes is well established. RTA catalyzes the hydrolysis of the *N*-glycosidic bond of a single adenine base within the sarcin–ricin loop (SRL) of the 28S rRNA, a conserved hairpin-like structure that interacts with eukaryotic elongation factors (EFs) 1 and 2, during ribosome translocation (1, 2, 9, 12, 13, 14, 15, 16). The active site of RTA consists of a large solvent-exposed cleft on one face of the molecule that accommodates adenine through a π-stacking network and hydrogen bonding (16, 17). Site-directed mutagenesis has identified the key residues within the active site that are associated with RNA *N*-glycosidase activity (18). Namely, Tyr-80 and Tyr-123 stabilize the incoming adenine base *via* a π-stacking network, whereas Arg-180 is involved in protonation of the adenine-leaving group. Glu-177 participates in the ultimate hydrolysis of the *N*-glycosidic bond. There is neither evidence that residues outside the active site properly contribute to SRL depurination nor are there any reports that RTA enzyme kinetics are subject to allostery.

That said, there is a growing body of evidence that SRL depurination is the second event in a two-step interaction between RTA and the ribosome (19, 20). According to the two-step model, RTA first associates with the C terminus of the acidic P-stalk proteins (RPLP1 and RPLP2; “P1” and “P2”) of the 60S ribosome before being guided to the SRL (20, 21, 22). Targeted mutagenesis identified residues along the face of RTA normally occluded by RTB as being critical for ribosomal recruitment, with Arg235 being the most important (23, 24). The structures of RTA in complex with C-terminal peptides of the P2 protein (“P2C11” and “P2C10”) were solved recently (25, 26). The structures revealed a mostly hydrophobic pocket on RTA formed by residues Tyr183, Arg235, Phe240, and Ile251 as the target of the P2 peptides. Short peptides corresponding to the P2 C terminus bind RTA with micromolar affinities (∼200–450 μM) and prevent RTA depurination of intact ribosomes, without interfering with the active site (27). Thus, the large hydrophobic face of RTA normally occluded by RTB is apparently “repurposed” in the cytoplasm to assist in recruitment to the ribosome.

In this study, we report the identification and characterization of 11 alpaca-derived single-domain antibodies (V_H_Hs) that recognize distinct epitopes on the hydrophobic surface of RTA normally occluded by RTB. We solved the cocrystal structures of four V_H_Hs (V9E1, V9F9, V9B2, and V9F6) bound to RTA, revealing that three of the four antibodies not only physically occlude the ribosomal P-stalk binding pocket on RTA but also mimic key P-stalk peptide interactions. The fourth V_H_H, V9F6, recognizes an epitope adjacent to but not overlapping the ribosomal P-stalk binding pocket. Only the V_H_Hs that directly obstruct the ribosomal P-stalk binding pocket neutralized RTA in cell-free translation assays and blocked RTA–P2 peptide interactions. Finally, we show that transient expression of V9E1 and other V_H_Hs as intracellular antibodies (“intrabodies”) renders Vero cells resistant to ricin toxin challenge to levels equivalent to that previously achieved with V_H_Hs targeting the active site of RTAs (28). Our findings support a model in which the interaction of RTA with ribosomal P-stalk proteins is a prerequisite to SRL depurination and raise the possibility of developing therapeutics that target two distinct sites on RTA.

## Results

### Identification of a collection of V_H_Hs that target the hydrophobic face of RTA normally occluded by RTB

As part of a V_H_H phage-display screening strategy targeting diverse epitopes on ricin toxin's A and B subunits (28, 29, 30, 31), we identified 11 V_H_Hs that recognized RTA but not ricin holotoxin (Table 1). The V_H_Hs grouped into five families based on complementarity-determining region 3 (CDR3) amino acid sequences (Table 1). The binding affinities of the 11 V_H_Hs for RTA ranged from potent to ultrapotent, as determined by surface plasmon resonance (SPR) (Table 1 and Fig. S1). V_H_Hs in families 1, 2, and 5 (n = 4 total) had equilibrium dissociation constants (*K*_*D*_) of ∼0.1 nM (0.073–0.22 nM; Table 1) with on (*k*_a_) and off (*k*_*d*_) rates ranging from 3.5 × 10^6^ to 6.5 × 10^6^ M^−1^ s^−^^1^ and 2.2 × 10^−4^ to 1.3 × 10^−3^ s^−1^, respectively. Six of the seven V_H_Hs in families 3 and 4 had ultrahigh affinities for RTA with estimated *K*_*D*_s of <0.03 nM, with on rates (*k*_a_) ranging from 3.2 × 10^6^ to 6.1 × 10^8^ M^−1^ s^−1^ and off rates (*k*_*d*_) too slow to be accurately measured by SPR with no dissociation observed up to 10 min after V_H_H–RTA complex formation. The seventh V_H_H (V9D12) had a *K*_*D*_ of ∼0.07 nM.

**Table 1:** Summary of V9 V_H_H families identified in this study

Family	V_H_H	Competition		*K*_*D*_ (nM)	CDR3	GenBank
		SyH7	JD4			
1	V9E1	−	−	0.11	AADRDRLPSAITYEYNY	MW389192
2	V9E5	+	−	0.075	AGDRDTTAQAMGLFGARGDY	MW389193
3	V9B2	+	−	<0.03	ATEEVCTLGIFGHGPDDY	MW389188
4	V9B8	+	−	<0.03	AAADPLPLICTEADEYNY	MW389189
4	V9D12	+	−	0.070	AAADPLPLICTEPDEYTY	MW389191
4	V9E9	+	−	<0.03	AAADPLPLICTEADEYNY	MW389194
4	V9F9	+	−	<0.03	AAADPLPLVCTWGDEYDY	MW389196
4	V9G11	+	−	<0.03	AAADPLPLLCTEADEYDY	MW389198
4	V9H10	+	−	<0.03	AAADPLPLICTEADEYNY	MW389199
5	V9F6	−	+	0.22	AAGSYAAILYAPSY	MW389195
5	V9D5	−	+	0.073	AAGSYAAILYAPSY	MW389190

Underline indicates V_H_Hs whose structures were solved in complex with RTA in this study.

The reactivity of the V_H_Hs with RTA but not ricin holotoxin suggested that the V9 series of V_H_Hs might recognize the face of RTA that is normally occluded by RTB. In support of this notion, the V_H_Hs failed to recognize a derivative of RTA (RVEc) that lacks the folding domain (residues 200–267) that normally associates with RTB (Fig. S2). Moreover, we performed competition ELISAs with two monoclonal antibodies (mAbs) (SyH7 and JD4) that recognize epitopes on opposite sides of the RTA–RTB interface (Table 1 and Fig. S3) (32). V9E1, the sole V_H_H in family 1, was not competed by either SyH7 or JD4, whereas V_H_Hs in families 2 (n = 1), 3 (n = 1), and 4 (n = 6) were competed by SyH7 but not JD4. The two V_H_Hs in family 5 were competed by JD4 but not SyH7. Thus, the five V_H_H families fall within three distinct competition groups that likely represent spatially distinct epitopes on the “underside” of RTA that is exposed only when RTA is liberated from RTB and translocated, across the ER membrane into the cell cytoplasm.

We next evaluated the 11 V_H_Hs for toxin-neutralizing activity (TNA) in a cytotoxicity assay in which ricin was mixed with each V_H_H across a range of antibody concentrations (0.01–1000 nM) and applied to Vero cells for 2 h. The cells were then washed to remove unbound antibody, and cell viability was measured 48 h later. As anticipated, none of the V9 V_H_Hs had any measurable TNA in this assay, even at >1000-fold molar excess over ricin. In contrast, V5E1, a previously described V_H_H with potent TNA, neutralized ricin with an IC_50_ of ∼5 nM (Fig. S2) (33). We conclude that the V9 V_H_Hs lack TNA in a conventional Vero cell cytotoxicity assay.

### Structural analysis of V_H_H–RTA complexes

**Figure 1:**
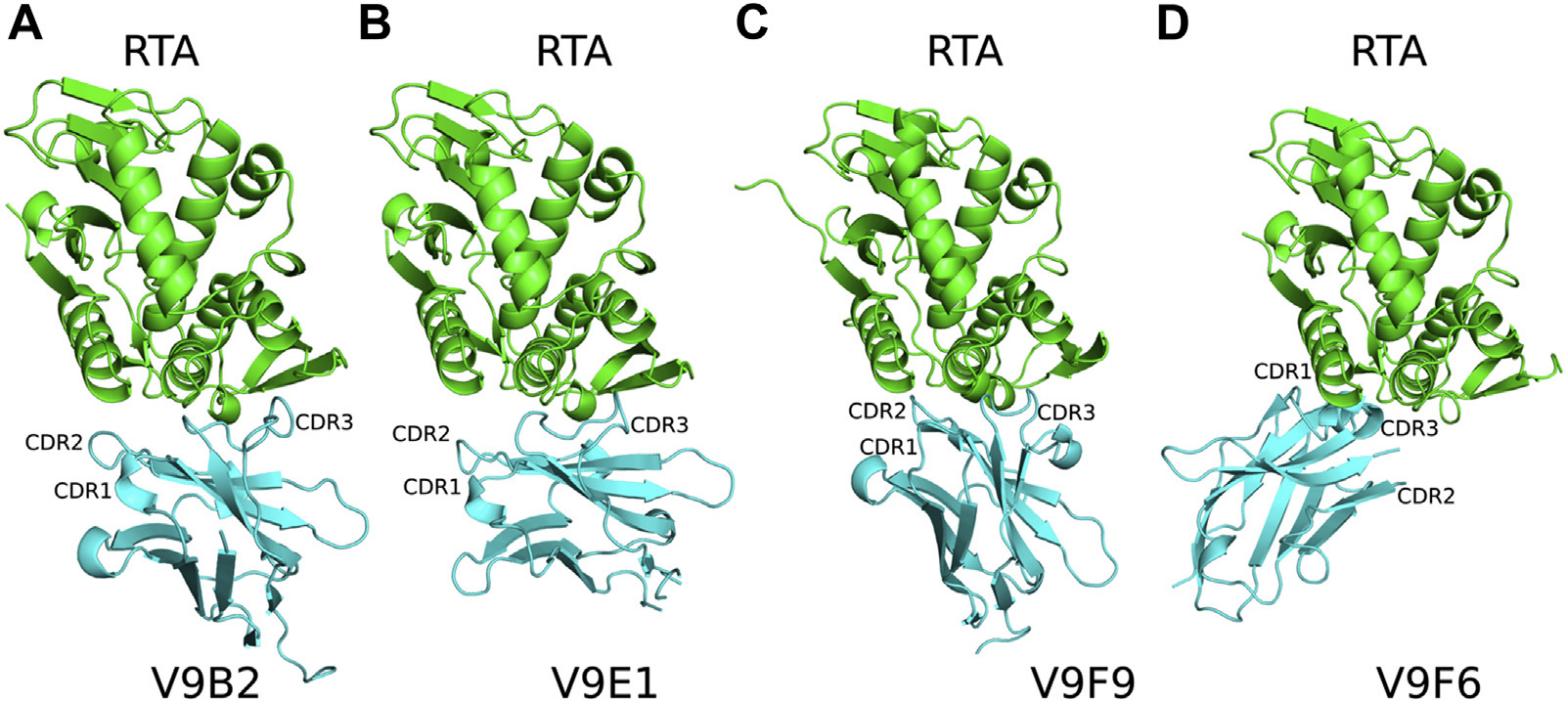
**Structures of V**_**H**_**H–RTA complexes.** Structures of RTA (*green*) in complex with (*A*) V9B2, (*B*) V9E1, (*C*) V9F9, and (*D*) V9F6 depicted as *ribbon diagrams*. Each V_H_H is colored *cyan*. RTA, ricin toxin subunit A.

We employed X-ray crystallography to identify the epitopes on RTA recognized by representative V_H_H family members. Toward this end, the structures of four V_H_Hs (V9E1, V9B2, V9F9, and V9F6) complexed with RTA were solved at resolutions ranging from 1.3 to 2.3 Å (Table 2 and S1 and S2). Each V_H_H assumed a typical immunoglobulin fold that consisted of nine β-strands arranged in two β-sheets comprised of five and four β-strands, with the exception of V9E1 as the first β-strand in V9E1's four β-strand sheet formed a loop instead of a β-strand. CDRs 1, 2, and 3 were located on one face of each V_H_H molecule, and each V_H_H antibody contained two, three, or four 3_10_ helices (Fig. 1). In all four V_H_H–RTA complexes, CDR elements 1 to 3 made contact with RTA, although the interaction was invariably dominated by CDR3. V_H_Hs V9B2, V9F6, and V9F9 exhibited the canonical disulfide bond between Cys-22 and Cys-96 (Cys-22 and Cys-95 in V9F9) linking FR1 and FR3 (34). The analogous disulfide bond was absent in V9E1, despite the presence of the requisite cysteine residues. V9B2 and V9F9 each had a second less common disulfide bridge between CDR2 with CDR3: residues Cys-50 and Cys-102 in V9B2 and Cys-49 and Cys-105 in V9F9. The structure of RTA in each complex consisted of seven α-helices (A–G), up to three 3_10_ helices (3_10a_–3_10c_), and 10 β-strands (a–j). Specific RTA secondary structure annotations are shown in Fig. S4 for reference.

**Table 2:** V_H_H–RTA binding data and interface information

V_H_H	*K*_*d*_^a^	EC_50_^a^	H-bonds^b^		SB^c^	SC^d^	Total BSA^e^
			Total	CDR1/2/3			
V9B2	<0.03	43.0	2	1/0/1	5	0.72	1897
V9F9	<0.03	19.1	5	1/3/1	7	0.70 (0.73^f^)	2028 (1994^f^)
V9E1	0.11	4.0	9	0/1/5	6	0.61	1864
V9F6	0.22	1.9	11	5/2/4	0	0.73	1130

a Nanometer, determined in *in vitro* translation assay.

b Hydrogen bonds.

c Salt bridges.

d Shape complementarity.

e Buried surface area.

f Second V_H_H–RTA complex in crystallographic asymmetric unit.

Among the four V_H_H–RTA structures solved, V9B2–RTA and V9E1–RTA were most similar to each other, as evidenced by an RMSD of 1.8 Å over 352 Cα atoms. By comparison, V9B2–RTA had an RMSD of 2.0 Å over 347 Cα atoms with V9F9–RTA, whereas V9E1–RTA and V9F9–RTA had an RMSD of 2.0 Å over 305 Cα atoms (Fig. S5). The most dissimilar structure was V9F6–RTA as V9F6 principally interacted with a structurally distinct RTA epitope. No conformational changes were evident in RTA when bound by antibody, as RTA in all four V_H_H–RTA complexes had RMSD values of 0.4 to 0.6 Å when all RTA Cα atoms were superimposed on RTA (Protein Data Bank [PDB] ID: 1RTC).

### Occlusion of ribosomal P-stalk binding site of RTA

**Figure 2:**
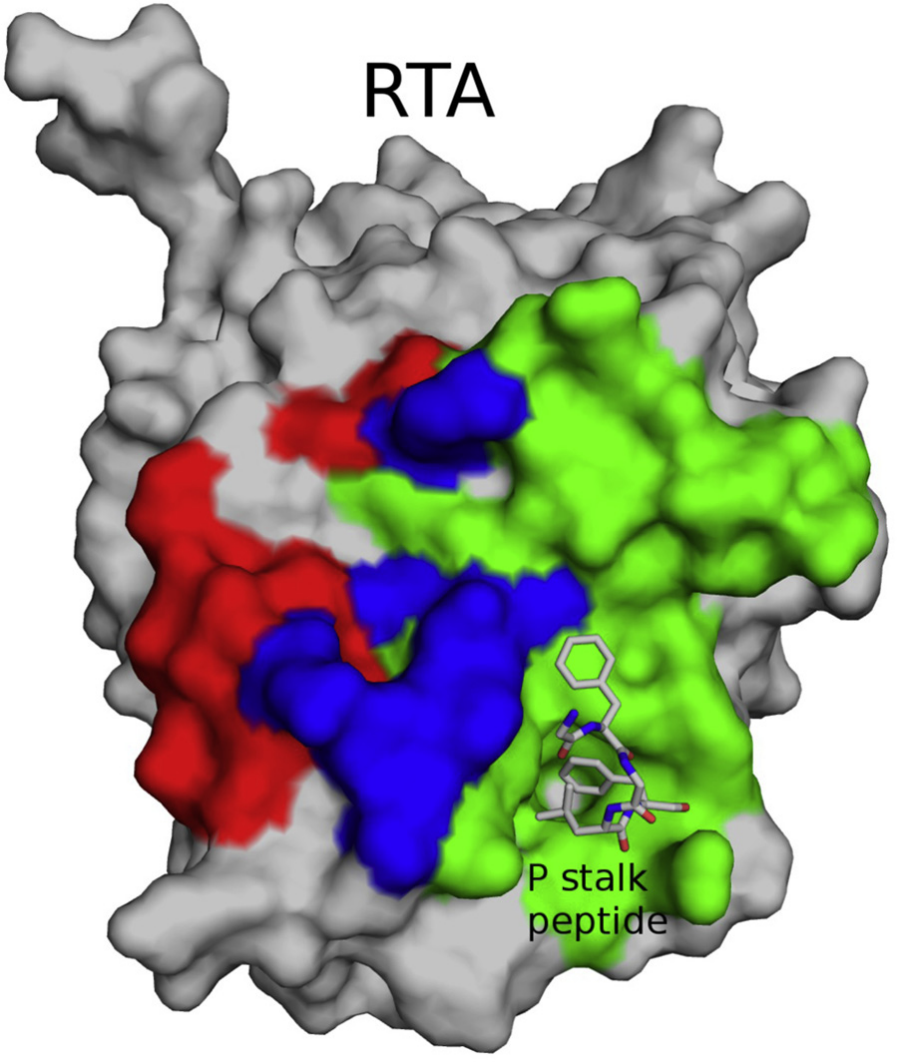
**Relative localization of V9F6, V9B2, V9E1, and V9F9 epitopes on RTA.** Molecular surface of RTA (*gray*) with the *red surface* highlighting the V9F6 epitope and the *green surface* depicting the combined epitopes of V9B2, V9E1, and V9F9. The *blue surface* depicts the overlapping epitopes of all four V_H_Hs. The P-stalk peptide highlighting the P-stalk binding site is drawn as *gray sticks* with carbon atoms *gray*, nitrogen atoms *blue*, and oxygen atoms *red*. RTA, ricin toxin subunit A.

Three of the four V_H_Hs (V9E1, V9B2, and V9F9), representing families 1, 3, and 4, recognized epitopes that overlap with the ribosomal P-stalk binding pocket of RTA (Figs. 2 and S5, *A* and *B*). Specifically, V9B2, V9E1, and V9F9 engaged RTA α-helix F (residues 183–194), β-strand i and j, and loop i–j (residues 232–236 and residues 240, 242, and 244) as well as helix 3_10_c (residues 246–249). V9E1 in addition contacts loop G–i on RTA (residue 223). The three V_H_Hs make slightly different contacts outside the P-stalk binding pocket, including with RTA α-helix A (residues 18, 19, 22–23, and 26), loop b–c (residue 41), loop E–F (residue 182), loop F–g (residue 196), α-helix G (residues 203 and 207), and several residues in helix 3_10_c (residues 250–251), and immediately C-terminal to helix 3_10_c (residue 253).

V9F6, in contrast, does not contact the P-stalk protein binding site (Figs. 2 and S5*C*). The epitope of V9F6 principally involves the C-terminal portion of α-helix A (residues 26, 29–30), loop–b (residues 34–37), β-strand b (residues 38–39), loop b–c (residues 40–41), β-strand c (residue 43), loop E–F (residue 182), and two residues in helix 3_10_c (residues 249–250) as well as residues immediately proximal to helix 3_10_c (residues 253, 261–262). That said, V9F6 does share a few contact points with the epitopes of V9B2, V9E1, and V9F9, including RTA α-helix A (residue 26), loop b–c (residue 41), loop E–F (residue 182), and residues 246, 249 to 250 within helix 3_10_c, along with residue 253 located immediately C-terminal to helix 3_10_c (Fig. 2).

### Detailed analysis of V_H_H interactions with the ribosomal P-stalk binding pocket of RTAs

**Figure 3:**
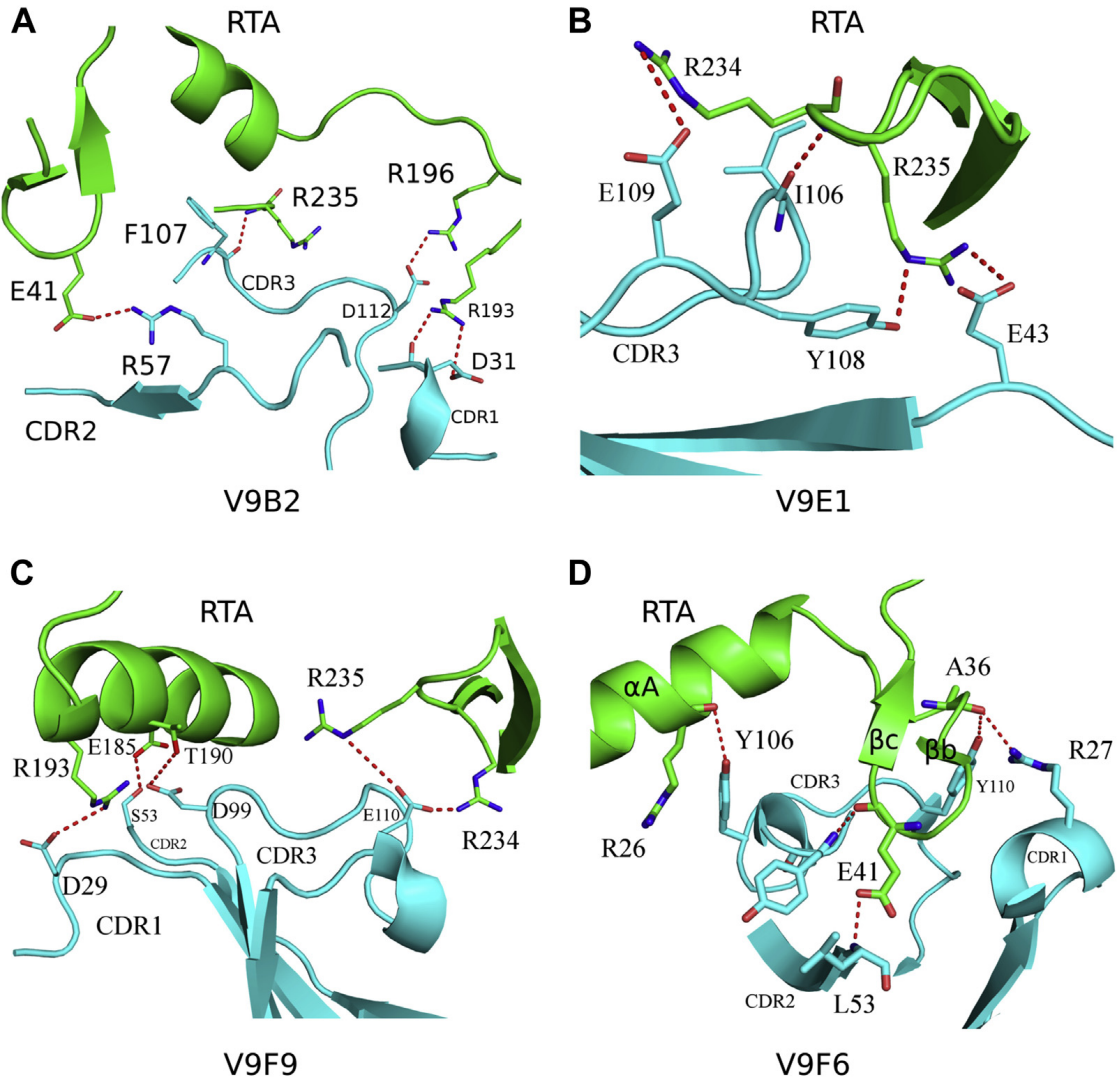
**Close-up of the V**_**H**_**H interactions with RTA.** Zoom in of the interface between the ribbon diagrams of RTA (*green*) in complex with (*A*) V9B2, (*B*) V9E1, (*C*) V9F9, and (*D*) V9F6 depicting key V_H_H contacts with RTA. Interacting V_H_H and RTA residues are drawn as *sticks* and color coordinated to their respective main-chain color with nitrogen atoms *blue* and oxygen atoms *red*. Hydrogen bonds and salt bridges are represented as *red dashes*. RTA, ricin toxin subunit A.

**Figure 4:**
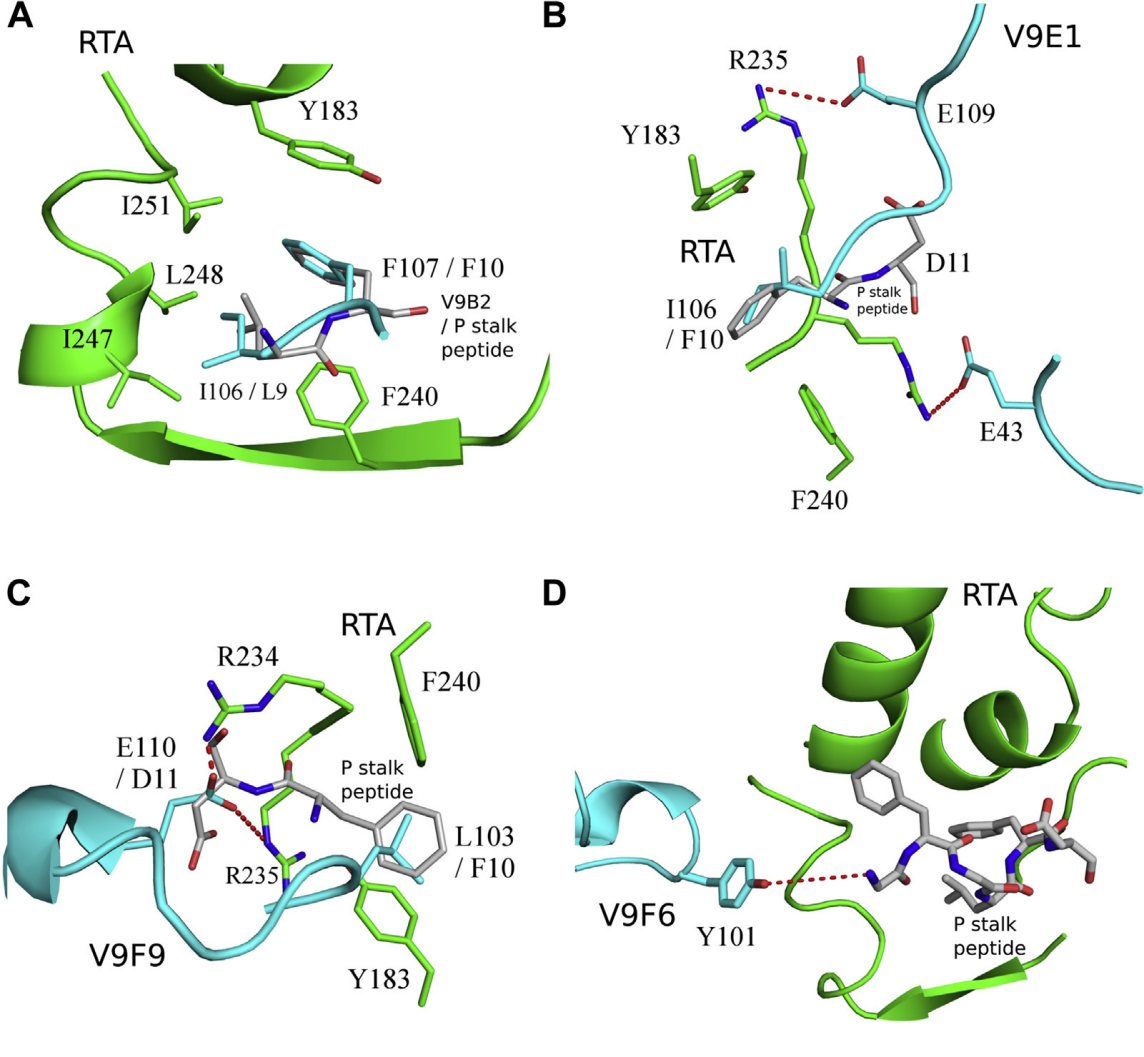
**V**_**H**_**H interactions with the P-stalk binding site.** Close-up view of the *ribbon diagrams* of RTA (*green*) in complex with (*A*) V9B2, (*B*) V9E1, (*C*) V9F9 depicting key V_H_H contacts with the P-stalk peptide, and (*D*) V9F6 highlighting the distant position of V9F6 relative to P-stalk binding site on RTA. Interacting V_H_H and RTA residues are drawn as *sticks* and color coordinated to their respective main-chain color with nitrogen atoms *blue* and oxygen atoms *red*. The P-stalk peptide is drawn as *gray sticks* with carbon atoms *gray*, nitrogen atoms *blue*, and oxygen atoms *red*. Hydrogen bonds and salt bridges are represented as *red dashes*. RTA, ricin toxin subunit A.

The interface between V9B2 and RTA buries 1897 Å^2^ and consists of two hydrogen bonds and five salt bridges (Table 2). The two hydrogen bonds form between the carbonyl oxygen atoms of Asp-31 in CDR1 and Arg-193 in RTA, along with the carbonyl oxygen atom from Phe-107 in CDR3 and Arg-235 of RTA. The five salt bridges occur between CDR1 Asp-31 and RTA Arg-193, CDR2 Arg-57 with RTA Glu-41, CDR3 Asp-100 with RTA Arg-189, and Arg-193 along with CDR3 Asp-112 with RTA-Arg196 (Fig. 3*A*). V9B2s CDR3 residue Phe-107 also forms a key π-stacking interaction between RTA residues Tyr-183 and Phe-240. This interaction mimics the π-stacking interaction that occurs between RTA residues Tyr-183 and Phe-240 with Phe-10 in the ribosomal P-stalk peptide (sequence SDDDMGFGLFD), as revealed in the RTA–P-stalk peptide crystal structure (Figs. 4*A* and S6*B*) (35). Further similarities include V9B2 CDR3 residue Ile-106 that burrows into a hydrophobic patch on RTA lined by Val-242, Ile-247, and Leu-248, in a similar fashion to Leu-9 within the P-stalk peptide in complex with RTA (Figs. 4*A* and S6*A*). V9B2 has a shape complementary (Sc) score of 0.72 with RTA.

The interface between V9E1 and RTA buries 1864 Å^2^ and involves nine hydrogen bonds and six salt bridges (Table 2). Two key hydrogen bonds are present between CDR3 residues Ile-106 and Tyr-108 with RTA residues Arg-234 and Arg-235, respectively. FR residue Glu-43 and CDR3 residue Glu-109 established two salt bridges with RTA residues Arg-234 and Arg-235, respectively (Fig. 3*B*). Arg-234 and Arg-235 are significant in that they form salt bridges with the C-terminal Asp residue in the P-stalk protein when bound to RTA (PDB ID: 5GU4) (Fig. S6*C*). V9E1 also inserts CDR3 residue Ile-106 between RTA residues Tyr-183 and Phe-240 similarly to P-stalk peptide residue Phe-10 (Fig. 4*B*). V9E1 has an Sc score of 0.62 with RTA.

The interface between V9F9 and RTA buries 2028 Å^2^ (1994 Å^2^ for the second V9F9–RTA complex within the crystallographic asymmetric unit) and consists of five hydrogen bonds and seven salt bridges, with contributions from each CDR. V9F9 CDR1 residue Asp-29 forms a salt bridge with RTA residue Arg-193, CDR2 residue Ser-53 H-bonded with RTA residue Glu-185, and CDR3 residue Asp-99 H-bonded with RTA residue Thr-190. Two salt bridges were also evident between V9F9 CDR3 residue Glu-110 and RTA residues Arg-234 and Arg-235 (Fig. 3*C*). As noted previously, Arg-234 and Arg-235 form key contacts with the P-stalk peptide residue Asp-11 (Fig. S6*C*). Finally, V9F9 CDR3 residue Leu-103 also mimics P-stalk peptide residue Phe-10 inserting between RTA residues Tyr-183 and Phe-240 (Fig. 4*C*). V9F9 has an Sc score of 0.70 with RTA (0.73 for the second V9F9–RTA complex within the crystallographic asymmetric unit).

V9F6 established a total of 11 hydrogen bonds between CDR1, CDR2, and CDR3 residues in V9F6 with RTA. Some of the key interactions included hydrogen bonding between the amide proton in CDR1 Arg-27 with the main-chain carbonyl oxygen of Ala-36 in RTA, the amide proton in CDR2 Leu-53 with Glu-41 in RTA, the amide proton in CDR3 Tyr-101 with the main-chain carbonyl oxygen of Glu-41 in RTA, and Tyr-110 with the main-chain carbonyl oxygen of Ala-36 in RTA (Fig. 3*D*). V9F6 does not contact the P-stalk protein binding site on RTA (Figs. 2 and S5*C*). In fact, the closest V9F6 residue Tyr-101 is 6.0 Å away from the N-terminal amine nitrogen atom of the superpositioned P-stalk peptide bound to RTA (Fig. 4*D*). Overall, the focal point of V9F6’s interaction with RTA centers on β-strands b and c, loop b and c, and α-helix A with the rest of the antibody angling away from V9B2, V9E1, and V9F9 when bound to RTA (Fig. S5). V9F6 has an Sc score of 0.73.

### Inhibition of RTA's RIP activity *in vitro*

The overlap between V9B2, V9F9, and V9E1’s epitopes with the ribosomal P-stalk binding site on RTA prompted us to examine whether the V_H_Hs interfere with the RIP activity of RTA. We were particularly interested in the comparison between V9E1 and V9F6 because the two V_H_Hs have similar binding affinities for RTA, but V9E1 blocks the P-stalk binding site on RTA, whereas V9F6 does not.

**Figure 5:**
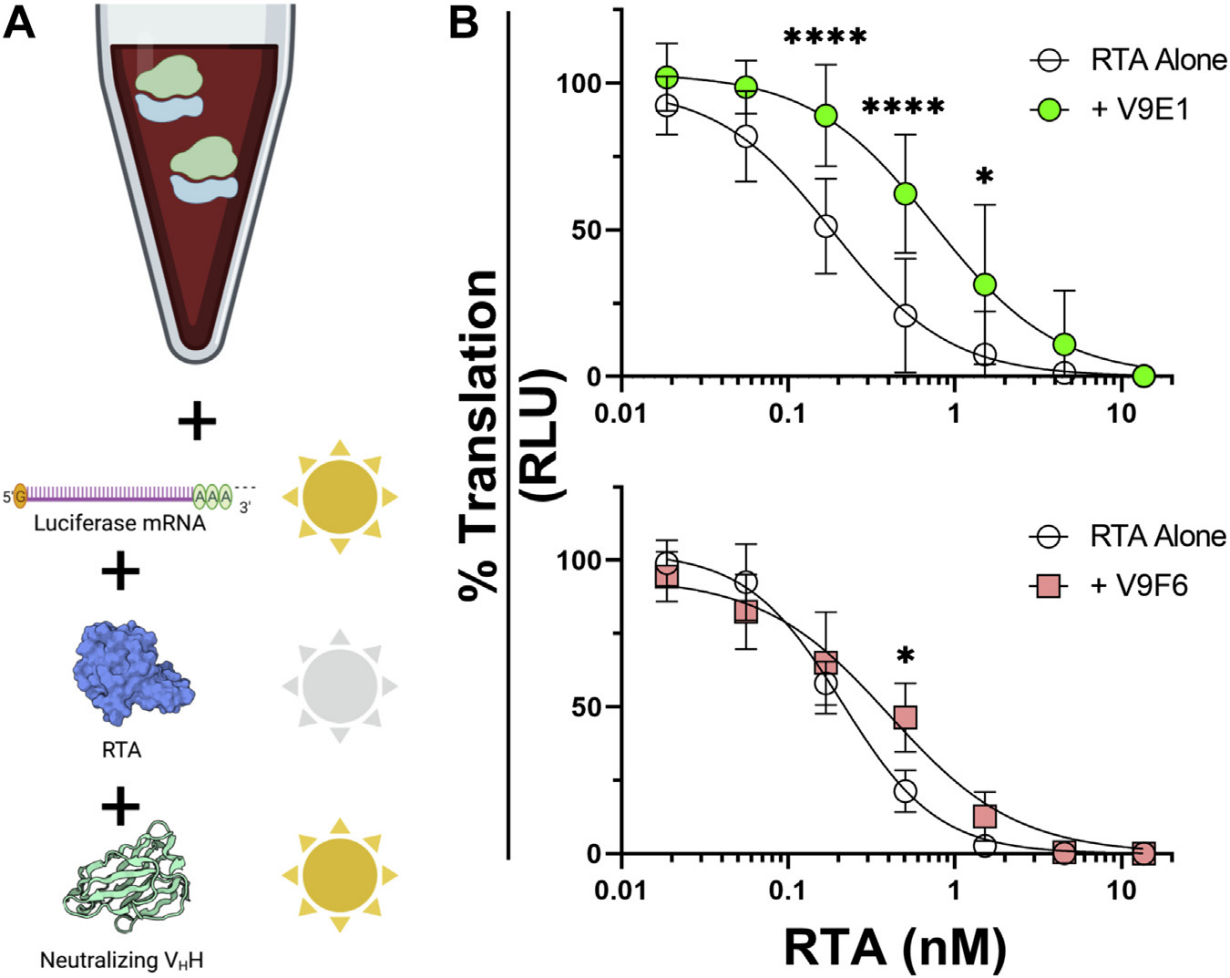
**Inhibition of RIP activity by V**_**H**_**Hs targeting the ribosomal P-stalk binding pocket of RTA.***A*, *in vitro* translation assays were performed by mixing 13.6 nM V_H_H with serial dilutions of RTA and then adding to a mixture containing rabbit reticulocyte lysate and luciferase mRNA. *B*, a standard curve (*open circles*) was established absent V_H_H addition. Shown are the results and nonlinear regression analyses of each V_H_H (*filled circles* or *squares*) normalized to positive controls without RTA added (100%). Data represent the mean ± SD of three (V9F6) or nine (V9E1) biological replicates. Significance was determined at each RTA concentration by two-way ANOVA. ∗∗∗∗*p* < 0.0001, ∗∗∗*p* < 0.001, ∗∗*p* < 0.01, ∗*p* < 0.05. RIP, ribosome-inactivating protein; RTA, ricin toxin subunit A.

To address this experimentally, the two V_H_Hs were assessed for their ability to neutralize RTA in an established *in vitro* translation (IVT) assay (28). Specifically, V9E1 and V9F6 were incubated with increasing amounts of RTA (0.02–13.6 nM) and then added to a master mixture containing mRNA template encoding firefly luciferase (Fig. 5*A*). In the absence of RTA, the reaction yielded >10^6^ relative light units, whereas the addition of RTA resulted in a dose-dependent reduction in translation, as evidenced by >1000-fold reduction in luciferase activity (Fig. S7). The addition V9F6 to the IVT assay had little to no effect on the RIP activity of RTA. In contrast, the addition of V9E1 (13.6 nM) resulted in a significant restoration of relative light unit, as compared with RTA treatment alone (Fig. 5*B*). In fact, V9E1’s activity was similar to that of V2A11, a high-affinity V_H_H (*K*_*D*_ = 0.31 nM) that occupies the active site of RTA (data not shown) (28). We conclude that V9E1 can indeed neutralize the RIP activity *in vitro* of RTA.

### Inhibition of RTA–P2 stalk recognition *in vitro*

**Figure 6:**
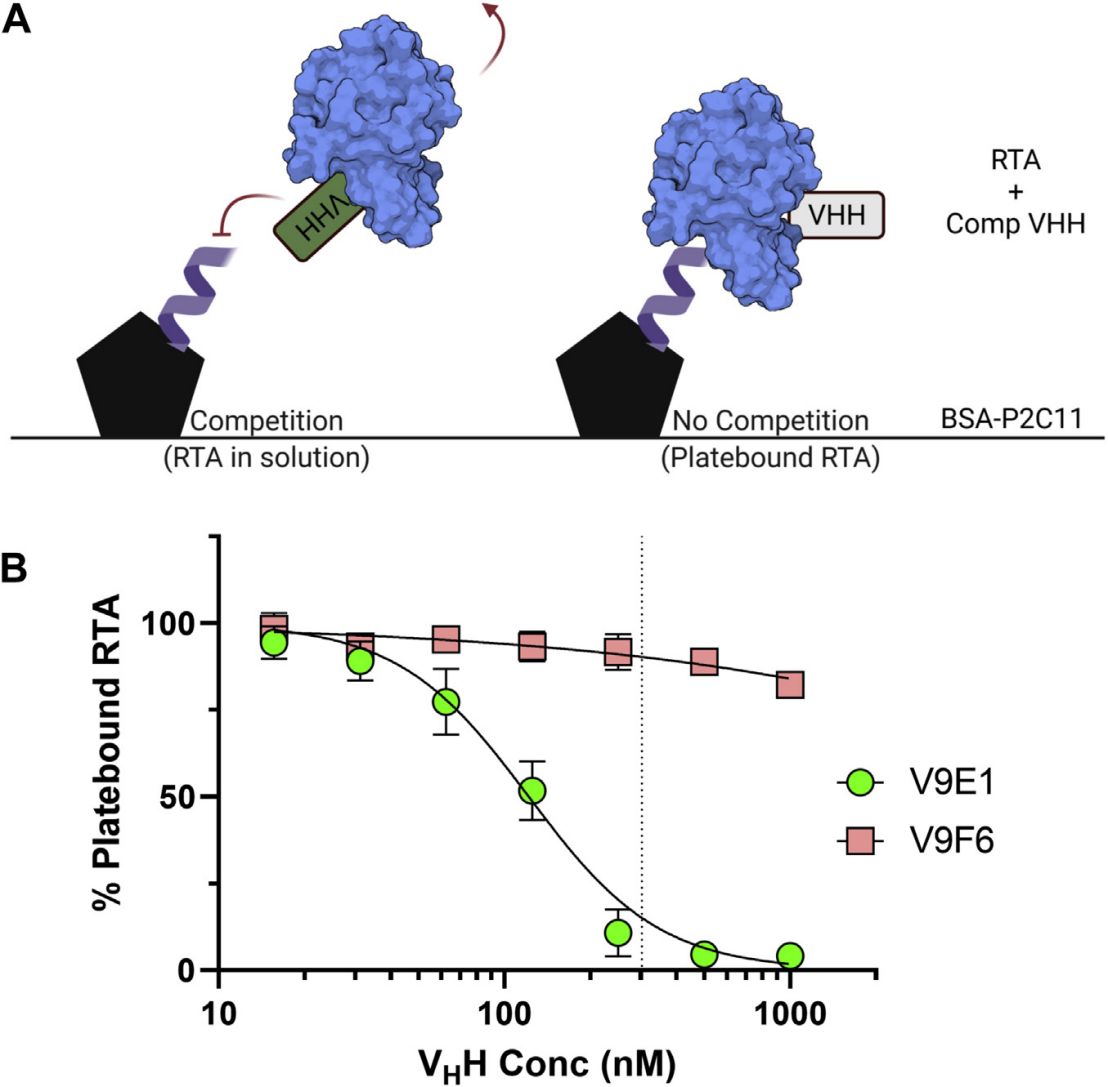
**V9E1 but not V9F6 prevents RTA-stalk binding.***A*, schematic of the RTA-stalk competition ELISA. *B*, RTA (303 nM) was incubated with serial dilutions of either V9E1 or V9F6 and added to BSA–P2C11-coated ELISA plates. Platebound RTA was detected with a noncompeting monoclonal antibody and normalized to control wells without V_H_H added (100%). *Vertical dotted line* indicates the 1:1 M ratio of RTA:V_H_H added to the plate. Each point represents the mean ± SD of three (V9F6) or four (V9E1) biological replicates. BSA–P2C11, P2C11 peptide–bovine serum albumin BSA conjugate; RTA, ricin toxin subunit A.

To test whether V9E1 interferes with the ability of RTA to associate with ribosome P-stalk proteins, we developed a solid phase binding assay in which microtiter plates were coated with a P2C11 peptide–bovine serum albumin BSA conjugate (BSA–P2C11) and then probed with RTA in the absence or the presence of antibody (Fig. 6). The addition of V9E1 resulted in a dose-dependent reduction of RTA attachment to BSA–P2C11 (Fig. 6). In fact, V9E1’s EC_50_ corresponded to a 2:1 RTA:V_H_H stoichiometry, whereas RTA binding to BSA–P2C11 was fully abolished at a 1:1 RTA:V_H_H stoichiometry. In contrast, V9F6 had no effect on the ability of RTA to interact with BSA–P2C11, even when the antibody was in molar excess. These results are consistent with predictions from the crystal structures in that V9E1 blocks access of RTA to the ribosomal P-stalk proteins, whereas V9F6, whose epitope is slightly offset vis a vis the ribosomal P-stalk binding site on RTA, has no effect.

### V9E1 intrabodies render Vero cells resistant to ricin intoxication

While V9E1 had no detectable ricin toxin neutralizing activity when applied to Vero cells extracellularly, we postulated that it might protect cells if expressed within the cell cytoplasm where it can encounter free RTA prior to engagement with the ribosome. In a recent study, we developed a protocol to successfully express RTA-specific V_H_Hs as intracellular antibodies or “intrabodies” in Vero cells (28, 36). Using that approach, the V9E1 and V9F6 coding sequences were cloned into the mammalian expression vector pcDNA3 and delivered into Vero cells by lipid nanoparticle (LNP) transfection. Both V9E1 and V9F6 were detected in cell lysates at comparable levels, as determined using an RTA-capture ELISA (Fig. S8).

**Figure 7:**
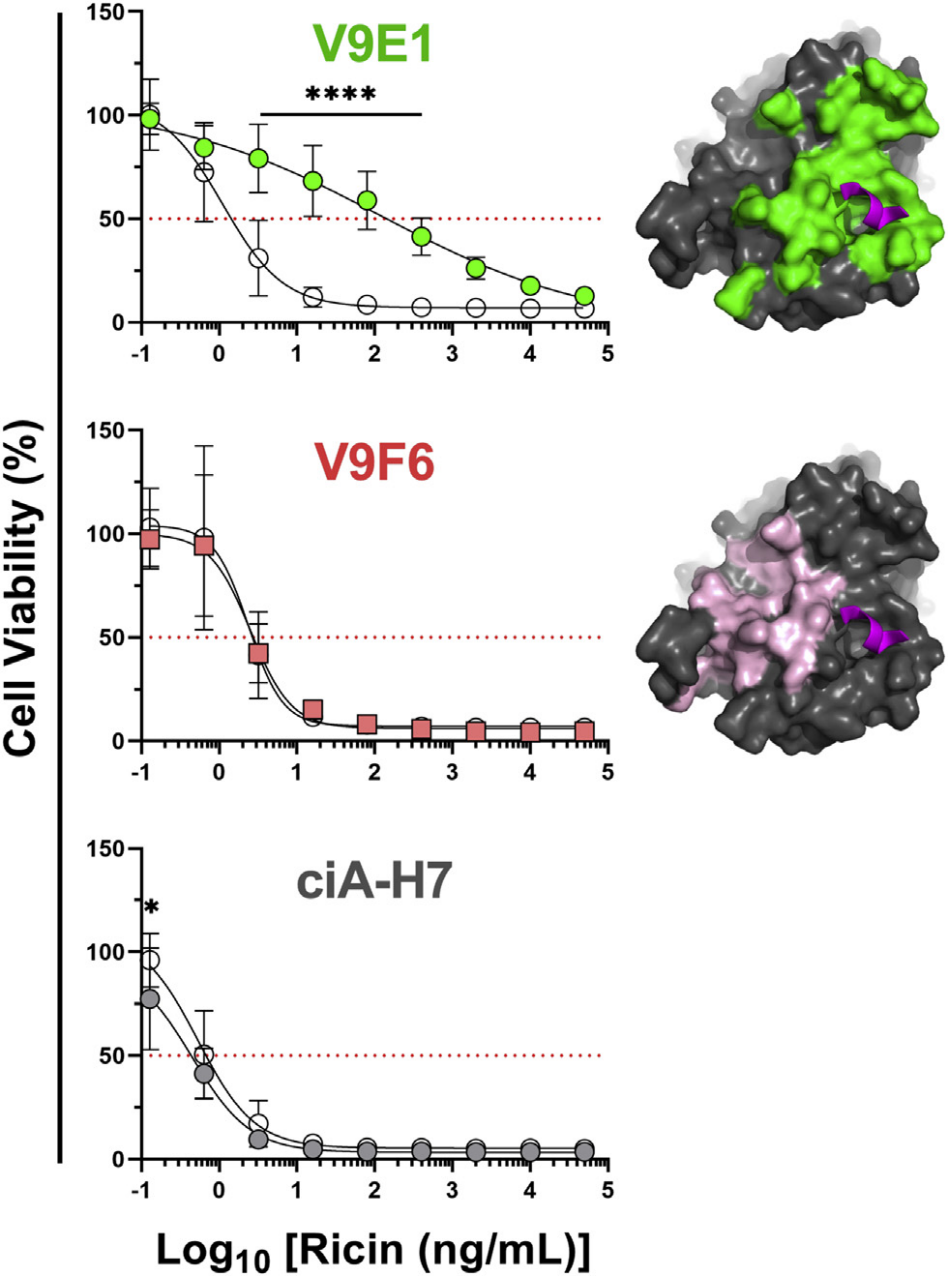
**Protection of Vero cells from ricin by intracellular V9E1.** Vero cells were seeded in 96-well plates for 1 day and then transiently transfected with V_H_H intrabodies. Cells were treated with dilutions of ricin 1 day after transfection, and viability was assessed 2 days post-treatment. *Left*, viabilities of cells transfected with vehicle control (LNP; *open circles*) or intrabodies (*filled circles* or *squares*) were measured as a percentage of “live” control cells not treated with ricin. Shown are the mean ± SD of at least three biological replicates representing three technical replicates each. In the absence of a BoNT-specific ELISA, expression of ciA-H7 intrabody was confirmed by Western blot (data not shown). Significance was determined at each ricin concentration by two-way ANOVA. ∗∗∗∗*p* < 0.0001, ∗∗∗*p* < 0.001, ∗∗*p* < 0.01, ∗*p* < 0.05. *Right*, epitopes on RTA highlighted for V9E1 (*green*) and V9F6 (*pink*) with the stalk peptide shown in *magenta*. BoNT, botulinum neurotoxin; LNP, lipid nanoparticle; RTA, ricin toxin subunit A.

To assess the impact of the V9E1 and V9F6 intrabodies on the sensitivity of Vero cells to ricin intoxication, transiently transfected cells were treated with escalating amounts of ricin toxin for 2 h, washed, and then assessed for viability 48 h later (Fig. 7). A botulinum neurotoxin–specific V_H_H, ciA-H7, was used as a control for these studies (37). Vero cells transfected with V9E1 were ∼100-fold more resistant to ricin, as compared with cells transfected with LNP. In contrast, Vero cells transfected with V9F6 remained as sensitive to ricin as control cells (Fig. 7). This result demonstrates that targeting the ribosomal P-stalk binding site of RTA is sufficient to protect the ribosomes from ricin’s RIP activity.

### Inactivation of RTA by the remaining V9 families of V_H_Hs

**Figure 8:**
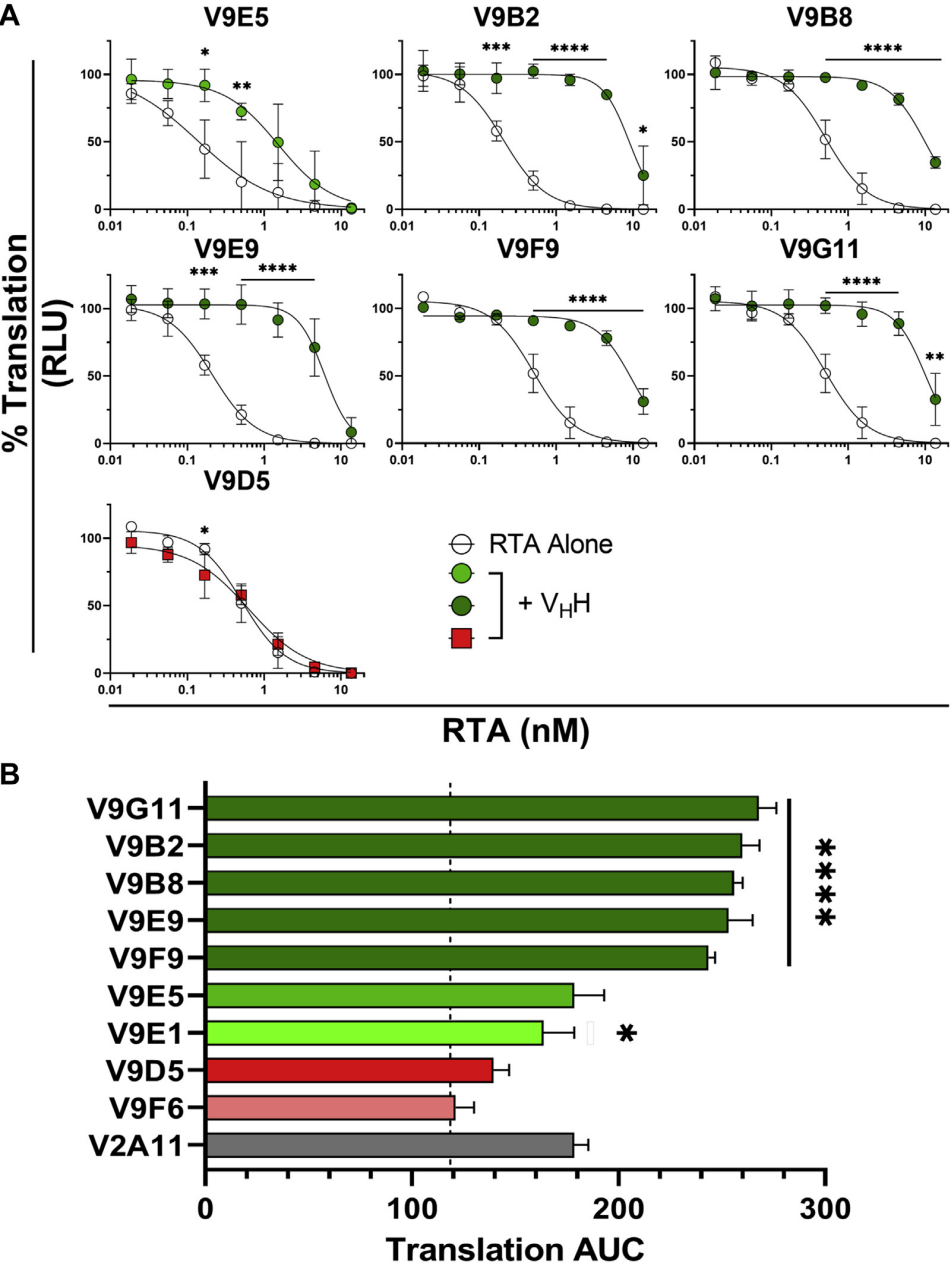
**Inhibition of the activity of RTA by V**_**H**_**H families 2, 3, and 4.***A*, *in vitro* translation assays were performed with V_H_Hs from families 2, 3, 4, and the remaining family 5 V_H_H (V9D5). Shown are mean ± SD of three replicate experiments. Significance at each RTA concentration was determined by two-way ANOVA. *B*, dose–response curves for each V_H_H–RTA combination were transformed, and area under the curve (AUC) analysis was performed. The *dashed line* represents the standard curve AUC (*x* = 118). The high-affinity active site V_H_H, V2A11, is also shown (*gray*). Significance was calculated using a one-way ANOVA with comparisons to the standard curve AUC shown. ∗∗∗∗*p* < 0.0001, ∗∗∗*p* < 0.001, ∗∗*p* < 0.01, ∗*p* < 0.05. RTA, ricin toxin subunit A.

We next turned our attention to the other V9 V_H_H family members (Table 1). V9B2 and V9F9 (families 3 and 4) were of particular interest because they occlude the ribosome P-stalk binding site of RTA like V9E1 but bind RTA with significantly higher affinity (Table 1 and Fig. 4). As predicted, V9B2 and V9F9 inhibited RTA from binding to P2C11 in a dose-dependent manner (Fig. S9). Three other V_H_Hs from family 4 (V9B8/V9E9/V9G11) also prevented RTA–P2C11 interactions, whereas V9D5, a member of family 5, did not (Fig. S9). Similarly, in the IVT assay, there was a clear hierarchy of RTA neutralization, with V9B2 and V9F9 and other V_H_Hs in family 4 (V9B8/V9E9/V9G11) being the most potent inhibitors, followed by V9E1 and V9E5 (families 1 and 2) (Fig. 8). In contrast, V9D5 (family 5) was ineffective at neutralizing RTA in the IVT assay. These results further demonstrate that direct obstruction of, and not just proximity to, the ribosomal recruitment site of RTA is necessary to block P2 stalk binding and to neutralize RTA RIP activity.

**Figure 9:**
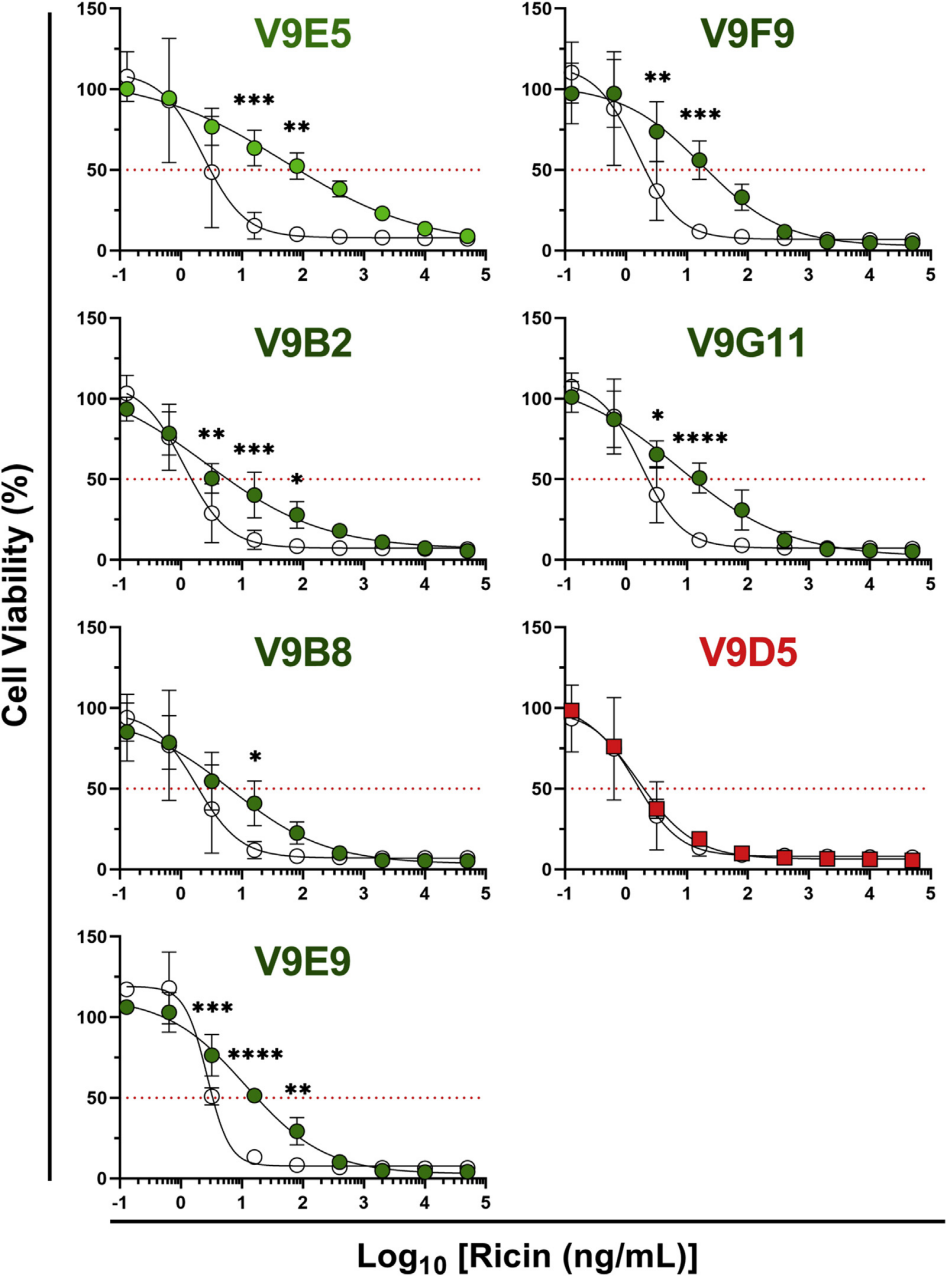
**Protection of Vero cells from ricin by family 2, 3, and 4 intrabodies.** Cytotoxicity assays were performed with intrabodies from families 2, 3, 4, and the remaining family 5 intrabody (V9D5). Viabilities of cells transfected with vehicle control (LNP; *open circles*) or intrabodies (*filled circles* or *squares*) were measured as a percentage of “live” control cells not treated with ricin. Shown are the mean ± SD of at least three biological replicates representing three technical replicates each. Significance was determined at each ricin concentration by two-way ANOVA. ∗∗∗∗*p* < 0.0001, ∗∗∗*p* < 0.001, ∗∗*p* < 0.01, ∗*p* < 0.05. LNP, lipid nanoparticle.

From these experiments, we expected that, when expressed as intrabodies, V9B2 and V9F9 and three other V_H_Hs in family 4 would be more effective than V9E1 at protecting Vero cells from ricin intoxication. To test this hypothesis, Vero cells were transiently transfected with pcDNA3 expression vectors encoding families 3 and 4 V_H_Hs (V9B2, V9B8, V9E9, V9F9, and V9G11) and then challenged with ricin toxin 1 day later. While each of the V_H_Hs rendered Vero cells more resistant to ricin than the mock-transfected cells, their activities were lower than anticipated considering their ultra–high-binding affinities (Fig. 9). In fact, area under the curve (AUC) analysis indicated that V9E1 (family 1) was superior to V_H_Hs in families 3 and 4 (data not shown). V_H_Hs in families 3 and 4 differ from V9E1 (family 1) in that they have (or are suspected to have) a second disulfide bond that tethers CDR2 and CDR3, which could reduce protein expression (Fig. S10) (34).

To examine this experimentally, Vero cells were transfected as noted previously, and then cell lysates were subjected to RTA ELISA. As predicted, V9B2 was expressed at low levels, as compared with V_H_Hs in families 1, 2, and 5 (Fig. S8). Indeed, all five V_H_Hs with a predicted second disulfide bond were expressed at low levels (Fig. S8). Nonetheless, V9B2-transfected cells were significantly more resistant to ricin, as compared with control cells, revealing that even small amount of antibody has the capacity to neutralize ricin (Fig. 9).

Finally, we evaluated V9E5 (family 2) and V9D5 (family 5) as intrabodies. Neither V9E5 nor V9D5 contain a second disulfide bond, and, correspondingly, both expressed well in transfected cells (Fig. S8). V9E5-transfected cells were significantly resistant to ricin compared with LNP cells (>22-fold increase in EC_50_), whereas V9D5 transfection conferred no protection from the toxin (Fig. 9).

### Correlation between epitope specificity and binding affinity in RTA inhibition

With the recent discovery of the P-stalk binding site and demonstration that preventing P-stalk binding reduces the enzymatic activity of RTA, there is interest in developing small molecules against RTA that target P-stalk binding site (38). Those approaches have yielded compounds with high specificity that mimic interactions of Phe-10 within the P2C11 peptide and RTA as revealed by structural analysis, similar to the mimicry we observed for V9B2, V9E1, and V9F9. These molecules are also capable of inhibiting the depurination activity of RTA and can reduce ricin cytotoxicity in a Vero cell model, albeit at high concentrations because of their low affinities for RTA. From our structural and functional analyses, we reasoned that the primary drivers for recruitment site–targeted neutralization are binding strength, proximity to the P-stalk binding site, and the ability to express intracellularly. With this in mind, we reasoned that a competition assay with V9B2, an ultra–high-affinity RTA binder that overlaps P2C6 binding on RTA, would enable accurate prediction of the neutralization strength of inhibitors targeting the P-stalk binding site.

In this assay, V9B2 was coated on the ELISA plate, and each potential competitor V_H_H was serially diluted and mixed with a fixed concentration of RTA (0.46 nM). There was a clear separation of V_H_H competition profiles into three clusters with unique binding site and strength differences, as predicted (Fig. S11). Ultra–high-affinity V_H_Hs in families 3 and 4 (V9B2, V9B8, V9E9, V9F9, and V9G11) competed strongly with V9B2 as evidenced by 50% binding inhibition at 1:1 V_H_H:RTA ratios. Next, V9E1 and V9E5, high-affinity V_H_Hs in families 1 and 2, competed more weakly with V9B2 for RTA with IC_50_ values at >25:1 V_H_H:RTA ratios. Conversely, neither of the high-affinity V_H_Hs in family 5 (V9F6 and V9D5) demonstrated notable competition, likely because of the fact that their epitopes are offset relative to the P-stalk binding pocket. AUC analyses of these data correlate strongly with cell-free RTA neutralization results using a simple linear regression model, showing an apparent delineation of these three clusters by both V9B2 competition and IVT results (Fig. S11). Multiple linear regression analysis accurately predicted intrabody-based ricin resistance from each of the nine V9 V_H_Hs examined. To correct for expression level differences, we incorporated V9B2 competition data as well as intrabody expression data into this analysis. We observed a strong correlation (*r*^2^ = 0.84) between both ELISAs and cytotoxicity results (Fig. S11). These results demonstrate that intracellular neutralization of the toxin by targeting the ribosomal recruitment event of RTA can be strongly predicted based on binding site specificity and affinity.

## Discussion

The efficiency by which RTA inactivates mammalian ribosomes is remarkable. The underlying chemistry involves hydrolysis of a single N-glycosidic bond of a conserved adenosine residue situated in the SRL of 28S rRNA (9). The catalytic center of RTA constitutes a solvent-exposed cleft on one side of the molecule that accommodates a protruding adenine base. While the molecular basis of the depurination reaction has been recognized for decades, only recently have we gained insight into how RTA is recruited to the ribosome in the first place (19, 24, 25, 39, 40, 41). Indeed, there is now compelling evidence that the RIP activity of RTA is actually a two-step event, wherein step 1 involves interactions with ribosome by P-stalk proteins before the second engagement with the SRL. The X-ray crystal structures of RTA in a complex with peptides derived from human P2 stalk protein revealed a putative ribosomal P-stalk binding pocket located on the opposite side of the molecule relative to the active site (25, 26, 35). Genetic and biochemical studies had already implicated this exact region of RTA as being important in associating with purified ribosomes (21, 41, 42). However, the importance of the P-stalk binding event within the context of intoxicated mammalian cells has remained unclear.

The results of our current study reveal that ribosome P-stalk binding is an essential step in the RIP activity of RTA. This conclusion is based on the demonstration that surgically targeting the ribosomal P-stalk binding pocket on RTA with high-affinity single-domain antibodies was sufficient to knock out the RIP activity of RTA in cell-free assays, as well as within the context of intoxicated cells. The comparison between two V_H_Hs, V9E1 and V9F6, proved especially informative. V9E1 and V9F6 have comparable binding affinities for RTA (0.11 and 0.22 nM, respectively) but slightly different epitopes relative to the P-stalk binding site. V9E1 occludes the P-stalk binding pocket and forms hydrogen bonds and π-stacking interactions with residues (*e.g.*, Arg234, Arg235, Tyr183, and Phe240) involved in P-stalk peptide recognition (25, 35). V9F6, by comparison, recognizes an epitope immediately adjacent to but not obstructing the P-stalk binding pocket. The relatively small difference in epitope location had significant consequences in terms of interfering with RTA activity: V9E1 inhibited RTA–P2C11 interactions, whereas V9F6 did not. V9E1 protected ribosomes from RTA in cell-free translation assays, whereas V9F6 did not. Finally, V9E1 conferred resistance to ricin toxin when expressed as an intrabody in Vero cells, whereas V9F6 did not. These results demonstrate that direct occlusion of the ribosomal P-stalk peptide binding site is sufficient to neutralize the RIP activity of RTA within the cell cytoplasm. Moreover, they suggest that ribosomal P-stalk protein interactions are likely confined to a relatively small surface area on RTA (*i.e.*, essentially outlined by the footprint of V9E1).

Moreover, blocking access to the P-stalk binding pocket of RTA attenuates the RIP activity of RTA to a degree equivalent to that achieved by interfering with the active site of RTA. This conclusion is based on the comparison of V9E1 with V2A11. V2A11 is one of seven V_H_Hs identified that target the active site pocket of RTA (28). The binding affinity of V2A11 for RTA is similar to V9E1 (0.11 nM *versus* 0.31 nM). The two antibodies have identical Sc scores (Sc = 0.61) and similar buried surface areas (1953 *versus* 1864 Å^2^). In the cell-free translation assays, V2A11 and V9E1 had identical RTA inhibition values, and, when comparing across studies of RTA, similar profiles in terms of conferring resistance to ricin toxin when expressed as intrabodies in Vero cells. In essence, docking with the ribosomal P-stalk proteins is a requisite step in the RIP activity, and as others have proposed, as important as the actual depurination event itself (21, 22, 42, 43).

In many ways, the two-step model, as put forth by Tumer *et al.* (21), explains the efficiency of RTA as a RIP. A longstanding question in the field is how RTA, following unfolding and translocation across the ER membrane, finds its substrate within the densely packed environment of the cell cytoplasm. Molecular chaperones like Hsc70 have been proposed to engage RTA following ER dislocation, whereas others have suggested that refolding may be facilitated by ribosomes themselves (44, 45). Irrespective of the how and where the folding event occurs, the sheer efficiency of RTA as a ribotoxin indicates that it wastes no time in accessing its substrate. Imai *et al.* (46) recently provided evidence that P-stalk proteins function as “factor-pooling platforms” that increase the local concentration of translational GTPases (“EFs”) near the ribosome surface to ensure optimal translation efficiency. Seemingly, RTA exploits (and competes for) the pathways used by EFs to engage with the GTPase-associated center and increase likelihood of encountering the SRL. This strategy may be conserved considering that other RIPs, such as Shiga toxin 2, have also been shown to bind to the C-terminal domain of ribosome P-stalk proteins (47).

While our focus up to this point has been on V9E1, it is just one of the nine V_H_Hs identified in this study capable of neutralization of RTA in the IVT assay and interfering with RTA–P2C11 association. Structural analysis of two of those additional V_H_Hs, namely V9B2 and V9F9, revealed occupancy of ribosome P-stalk binding pocket of RTA in a manner similar to V9E1. Six of the nine V_H_Hs (including V9B2 and V9F9) are remarkable in that their binding affinities for RTA were estimated to be in the low picomolar range (<0.03 nM). With extremely slow off-rates, V9B2 and V9F9 (and the other V_H_Hs) were expected to be exceptionally potent inhibitors of ricin toxin when expressed as intrabodies. While they did render Vero cells more resistant to ricin than controls, the levels were not commensurate with binding affinities. As it turns out, V9B2 and V9F9 (as well as others) expressed poorly as intrabodies, most likely because of the fact that they carry a second noncanonical disulfide bond between CDR2 and CDR3. Indeed, it is generally recognized that V_H_Hs and single-chain variable fragments carrying a second disulfide bond are poorly expressed because of the reducing environment of the cytoplasm (48, 49). Our preliminary efforts to engineer out the pertinent cysteine residues in V9B2 and V9F9 using methodologies described by others have proven unsuccessful to date (T. Czajka, unpublished results) (49, 50).

The remarkably high-binding affinities reported here for six of the V9 V_H_Hs are certainly unusual but not unprecedented in the case of ricin toxin or other agents (33). For example, several V_H_Hs with picomolar affinities for the spike protein of severe acute respiratory syndrome coronavirus 2 were isolated from immune camelid libraries (51, 52). There are likely multiple factors that give rise of such tight binders, including a hydrophobic target interface and the addition of noncanonical disulfide bonds within CDR elements that minimize the entropic cost of antigen engagement (53, 54). The relative binding affinities of V_H_Hs can be further enhanced through oligomerization as dimers, trimers, or even higher order oligomers (55, 56, 57). Toward this end, we have found that the potency of V9E1–V2A11 heterodimers greatly exceeds that of any single V_H_H monomer tested to date (T. Czajka and N. Mantis, manuscript in preparation). Such constructs if delivered by mRNA technologies may offer an entirely new strategy for ricin toxin postexposure therapeutics (58).

## Experimental procedures

### Chemicals and biological reagents

Ricin toxin (*R. communis* agglutinin II; RCA_60_) and RTA were purchased from Vector Laboratories and dialyzed against PBS to remove residual sodium azide prior to use in cytotoxicity assays. Unless noted otherwise, all reagents were purchased from Sigma–Aldrich.

### V_H_H ELISAs

Anti-RTA mAbs were coated in 96-well plates at a concentration of 1 μg/ml in PBS overnight at 4 °C. Plates were then blocked with 2% goat serum in PBS containing 0.1% Tween-20 (PBST) for 2 h at room temperature. Ricin, RTA, or RVEc was then applied to the plates at 1 μg/ml to be captured by the anti-RTA antibodies for 1 h, and unbound protein was washed away with PBST. V_H_Hs were then applied at a concentration of 330 nM in PBS for 1 h. Unbound V_H_H was washed away with PBST, and bound V_H_H was detected for 1 h with an anti-E-horseradish peroxidase (HRP) secondary antibody (Bethyl Labs). 3,3′,5,5′-Tetramethylbenzidine (TMB; SureBlue; SeraCare) was used to develop the plates and was then quenched with 1 M phosphoric acid. Absorbance at 450 nm was read on a VersaMax microplate reader (Molecular Devices).

### Cloning, expression, and protein purification for structural analysis

The PCR amplicons for the four V_H_Hs were subcloned into the pSUMO expression vector encoding an N-terminal decahistidine and SUMO tag. RTA (residues 1–268) was subcloned into the N-terminally decahistidine-tagged MCSG7 expression vector. All clonings were performed using a standard ligase-independent cloning protocol. All V_H_Hs and RTA were expressed in *Escherichia coli* strain BL21(DE3)-pRARE. The transformed bacteria were grown at 37 °C in terrific broth medium and induced at 20 °C with 0.1 mM (IPTG) at an absorbance at 600 nm of 0.6 for ∼16 h at 20 °C. After induction, cells were harvested and resuspended in 20 mM Hepes (pH 7.5) and 150 mM NaCl. The cell suspension was sonicated and centrifuged at 30,000*g* for 30 min. After centrifugation, the protein-containing supernatant was purified by nickel-affinity and size-exclusion chromatography on an AKTAxpress System (GE Healthcare), which consisted of a 1 ml nickel affinity column followed by a Superdex 200 16/60 gel filtration column. The elution buffer consisted of 0.5 M imidazole in binding buffer, and the gel filtration buffer consisted of 20 mM Hepes (pH 7.5), 150 mM NaCl, and 20 mM imidazole. Fractions containing each V_H_H and RTA were pooled and subject to tobacco etch virus protease cleavage (1:10 weight ratio) for 3 h at room temperature in order to remove their respective fusion tags. The cleaved proteins were passed over a 1 ml Ni–NTA agarose (Qiagen) gravity column to remove tobacco etch virus protease, cleaved residues, and uncleaved fusion protein. To generate each V_H_H–RTA protein complex, RTA was mixed with each V_H_H in a 1:1 stoichiometry then concentrated to 10 mg/ml.

### Crystallization and data collection

V_H_H–RTA crystals were grown by sitting drop vapor diffusion at 20 °C using a protein to reservoir volume ratio of 1:1 with total drop volume of 0.2 μl. Crystallization solutions are shown in Table S1. Crystals were flash frozen in liquid nitrogen after a short soak in the respective crystallization buffers supplemented with 25% ethylene glycol. Data were collected at the 24-ID-C and 24-ID-E beamlines at the Advanced Photon Source, Argonne National Labs. All data were indexed, merged, and scaled using HKL2000 (59) and then converted to structure factors using CCP4 (60).

### Structure determination and refinement

The V_H_H–RTA complex structures were solved by molecular replacement using the program Phaser (61). Molecular replacement calculations were performed using the coordinates of the ricin A chain as a search model for RTA (PDB ID: 1RTC) in all four V_H_H–RTA complexes. The V_H_H coordinates used as a search model for all four V_H_H–RTA complexes were D10 (PDB ID: 4LGR) or A9 (PDB ID: 6CWG) with all three of the CDRs removed from the search model. The resulting phase information from molecular replacement was used to autobuild the polypeptide chain of the V_H_H within each complex using automated refinement procedure (62). Further manual model building was performed with the open source software, Coot (63), combined with structural refinement employing the PHENIX package (64). Data collection and refinement statistics are listed in Table S2. Molecular graphics were prepared using PyMOL (Schrodinger, DeLano Scientific LLC).

### P2C11 peptide competition

To evaluate V_H_H/ribosomal stalk competition for RTA binding, 96-well ELISA plates (Immulon 4HBX) were coated overnight at 4 °C with 10 μg/ml of BSA–P2C11 (Genemed Synthesis, Inc) in PBS. Plates were blocked with PBS containing goat serum (2% v/v) and Tween-20 (0.1% v/v) for 2 h at room temperature. During block, RTA (303 nM) was incubated with twofold serial dilutions of V_H_H in 96-well dilution plates for 30 to 60 min with 12 wells per plate receiving only RTA and block buffer to serve as 100% binding controls. Following block, V_H_H–RTA samples were transferred to the ELISA plates for 1 h. 1 μg/ml PB10 (anti-RTA mAb) (reference) was added for 1 h followed by HRP-conjugated goat antimouse immunoglobulin G secondary antibody (1:2000 dilution; SouthernBiotech) for 30 min. TMB (SureBlue; Kirkegaard & Perry Labs) was added for 6 to 10 min followed by stop solution (1 M phosphoric acid). ELISA plates were analyzed using a SpectraMax iD3 spectrophotometer equipped with Softmax Pro 7 software (Molecular Devices) at an absorbance at 450 nm. Unbound RTA, PH12, and 2° antibody were removed after each incubation period by washing plates with PBS-Tween (0.1%). RTA, V_H_Hs, and antibodies were diluted in block buffer.

### V9E1 and V9B2 competition assays

96-Well ELISA plates were coated overnight at 4 °C with 1 μg/ml of V9E1 or V9B2 in PBS. Competition ELISAs were performed as described for the stalk competition, with the exception of the RTA and V_H_H concentrations. During block, RTA (4.6 nM for V9E1 competition or 0.46 nM for V9B2 competition) was incubated with threefold serial dilutions of V_H_H in 6-well dilution plates for 30 to 60 min with 12 wells per plate receiving only RTA and block buffer to serve as 100% binding controls.

### Intrabody detection

Intrabody detection ELISAs were performed as described previously with minor alterations (28). 96-Well ELISA plates were coated overnight at 4 ^°^C with the mAb PH12 (1 μg/ml in PBS) (65). Plates were washed and blocked for 2 h at room temperature. Following block, RTA (1 μg/ml in PBS) was applied to ELISA plates for 1 h. Transfected cell lysate was serially diluted in duplicate and added to plates for 1 h. Plates were washed and HRP-conjugated anti-E-tag antibody (1:10,000 dilution; Bethyl Laboratories; catalog no.; A190-132P) was applied for 1 h. Plates were washed, and 100 μl TMB was added for 5 to 10 min followed by stop solution. ELISA plates were analyzed using a SpectraMax iD3 spectrophotometer equipped with Softmax Pro 7 software at an absorbance at 450 nm. Purified V_H_H protein with a C-terminal E-tag was used as a positive binding control for each transfection. Plates were washed following each step in PBS-Tween (0.1%). Cell lysates and secondary antibody were diluted in block buffer.

### *In vitro* RTA inhibition

V_H_Hs at a stock concentration of 218.2 nM in dimethyl sulfoxide were diluted in 1% BSA in PBS (w/v) and mixed with threefold serial dilutions of RTA in PBS from 54.5 to 0.07 nM, to a final concentration of 13.6 nM V_H_H and 13.6 to 0.019 nM RTA. This was added to an ice-cold mixture containing luciferase mRNA (3.7 μg/ml; Ambion, Inc) and Retic Lysate IVT Kit (Thermo Fisher Scientific). The cocktail was incubated for 90 min at 30 °C and then chilled on ice for 5 min before being transferred to wells of an opaque 96-well microtiter plate with an equal volume (20 μl) of Bright-Glo luciferase substrate (Promega) at room temperature. Luminescence was detected immediately using a SpectraMax iD3. Luciferase translation was determined as a percentage of positive control reactions without RTA added. Standard curves were generated for RTA dilutions with dimethyl sulfoxide, and no V_H_H was added.

### Vero cell culture, transfection, and lysis

Vero cells (American Type Culture Collection) were cultured in Dulbecco’s minimal essential medium with fetal bovine serum (10% v/v) and penicillin/streptomycin at 37 °C (5% CO_2_). For ELISAs and Western blotting, cells were transfected in 6-well plates and lysed 48 h after transfection, as described previously (28). For cytotoxicity assays, cells were transfected in 96-well plates and treated with ricin 24 h after transfection, as described (28). Viability was determined using Cell Titer-Glo (Promega) and a SpectraMax iD3 for luminescence detection. Viability was measured as a percentage of live control cells (transfected but not treated with ricin).

### Statistical analysis

Statistical analyses were performed using GraphPad Prism 9.1 software (GraphPad Software, Inc) for Windows. For IVT assays, a one-way ANOVA was with the Tukey's post hoc test to compare areas under the curve for each condition, and a two-way ANOVA was used with the Sidak post hoc test to compare the percent translation between V_H_H and the RTA standard curves at each RTA concentration. For intrabody ELISA expression analysis, a one-way ANOVA with the Tukey's post hoc test was used to compare each transfection condition. For intrabody cytotoxicity results, a two-way ANOVA was used with the Sidak post hoc test to compare transfected and LNP-vehicle control cell viabilities at each ricin concentration. A simple linear regression and Pearson correlation analysis were performed between the AUC data for competition ELISAs and IVT dose–response curves. To predict intrabody-based Vero cell protection, a multiple linear regression least-squares analysis was performed with cytotoxicity AUC as the dependent outcome and intrabody ELISA and V9B2 competition areas under the curve as independent variables.

### PDB accession numbers

The structures generated in this study were deposited in the PDB (http://www.rcsb.org/pdb/) under accession numbers 7TGF for V9B2–RTA, 7TGI for V9E1–RTA, 7TH3 for V9F6–RTA, and 7TH2 for V9F9–RTA as described in Table S1.

## Data availability

All data associated with the results presented in this article are included herein or within the supporting information.

## Supporting information

This article contains supporting information.

## Conflict of interest

The authors declare that they have no conflicts of interest with the contents of this article.
