# Supplementary material for: Single-domain antibodies neutralize ricin toxin intracellularly by blocking access to ribosomal P-stalk proteins
Source: J Biol Chem. 2022 Feb 17;298(4):101742. doi: 10.1016/j.jbc.2022.101742 (PMC8941211; doi:10.1016/j.jbc.2022.101742)
Supplement: Supplemental Figures S1–S12, Tables S1 and S2 [file mmc1.pdf]

SUPPORTING INFORMATION

Table S1. V_HH-RTA crystallization solutions

**V_HH**	**solutions**
V9B2	100 mM sodium citrate pH 4.0, 30% PEG 6,000, and 1 M lithium chloride
V9E1	200 mM magnesium chloride, 20% PEG 3,350.
V9F6	418 mM ammonium chloride, 22% PEG 3,350.
V9F9	100 mM MES pH 6.5, 200 mM sodium thiocyanate, 8% dioxane, 1,740 mM ammonium sulfate

Table S2. Data collection associated with X-ray crystal structures				
**Data Collection**				
Complex	V9B2-Ricin	V9E1-Ricin	V9F6-Ricin	V9F9-Ricin
d_{min} (Å)	1.3	2.1	2.3	1.8
No. of reflections	3402493	310176	664531	2349514
Average redundancy[a]	6.3(4.6)	1.7(1.5)	1.7(1.7)	3.1(3.1)
$(I)/(\delta)$[a]	34.4(0.9)	16.6(1.3)	21.2(4.6)	25.7(3.5)
Completeness[a] (%)	100(100)	98.7(97.0)	98.6(99.6)	98.0(96.8)
R_{merge}[a,b] (%)	7.4(208.4	9.1(68.2)	4.0(17.7)	11.5(90.7)
CC1/2[a,c]	(0.52)	(0.59)	(0.96)	(0.84)
**Refinement**				
Bragg spacings (Å)	49.9-1.3	42.2-2.1	47.8-2.3	47.4-1.8
Space group	$P4_12_12$	C2	$P2_1$	$P2_1$
Cell parameters: *a,b,c* (Å) / α,β,γ (°)	50.8, 50.8, 260.0	44.1, 84.4, 109.9 / β = 97.0	38.3, 67.1, 68.1 / β = 91.0	60.6, 63.8, 99.3 / β = 107.1
R[d] / R_{free}[e] (%)	15.8 / 18.1	17.6 / 22.2	24.4 / 27.3	14.9 / 18.7
No. of reflections	76949	22926	15296	61585
No. of waters	399	120	280	542
Rmsd bond length (Å)	0.011	0.008	0.004	0.008
Rmsd bond angle (°)	1.102	0.871	0.679	0.911
Ramachandran favored / allowed[f] (%)	98.9 / 100	97.5 / 100	96.7 / 100	98.8 / 100
PDB code	-	-	-	-

FIGURE S1

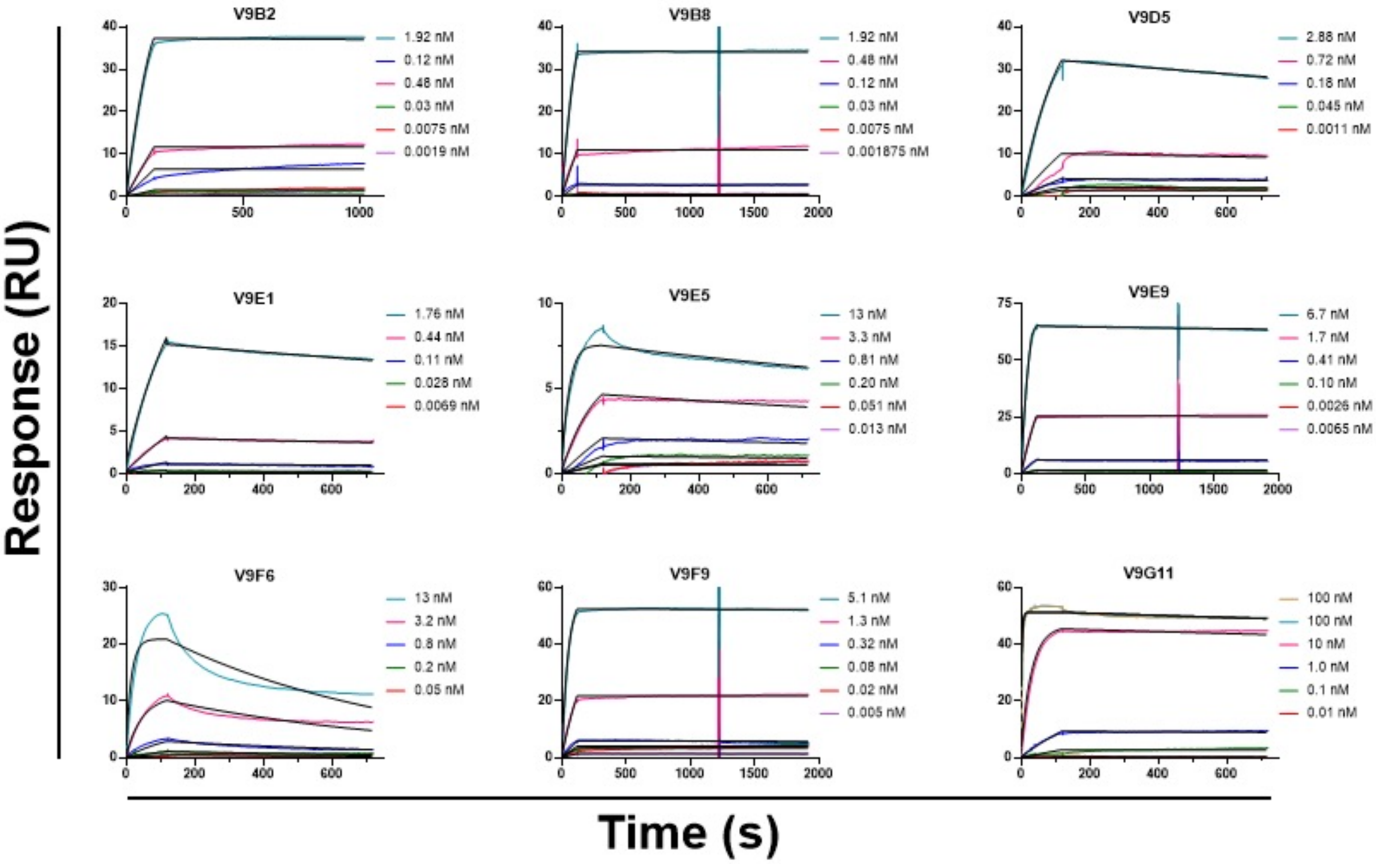

Figure S1. SPR analysis of V_HH binding to RTA. Rabbit polyclonal anti-E-tag antibodies were covalently linked to a series S CM5 sensor chip using a Biacore T200. Each of the nine V_HHs was covalently captured via C-terminal E-tags up to a maximum of 100 RU on series S CM5 sensor chips using a Biacore T200. RTA was injected at the indicated concentrations. Shown is the mean of 2-3 replicate experiments. Colored lines represent experimental data, whereas black lines represent the mean fit as determined using a 1:1 Langmuir binding model. V9F9 was performed in duplicate. V9G11 is a single replicate at a broad concentration range.

FIGURE S2

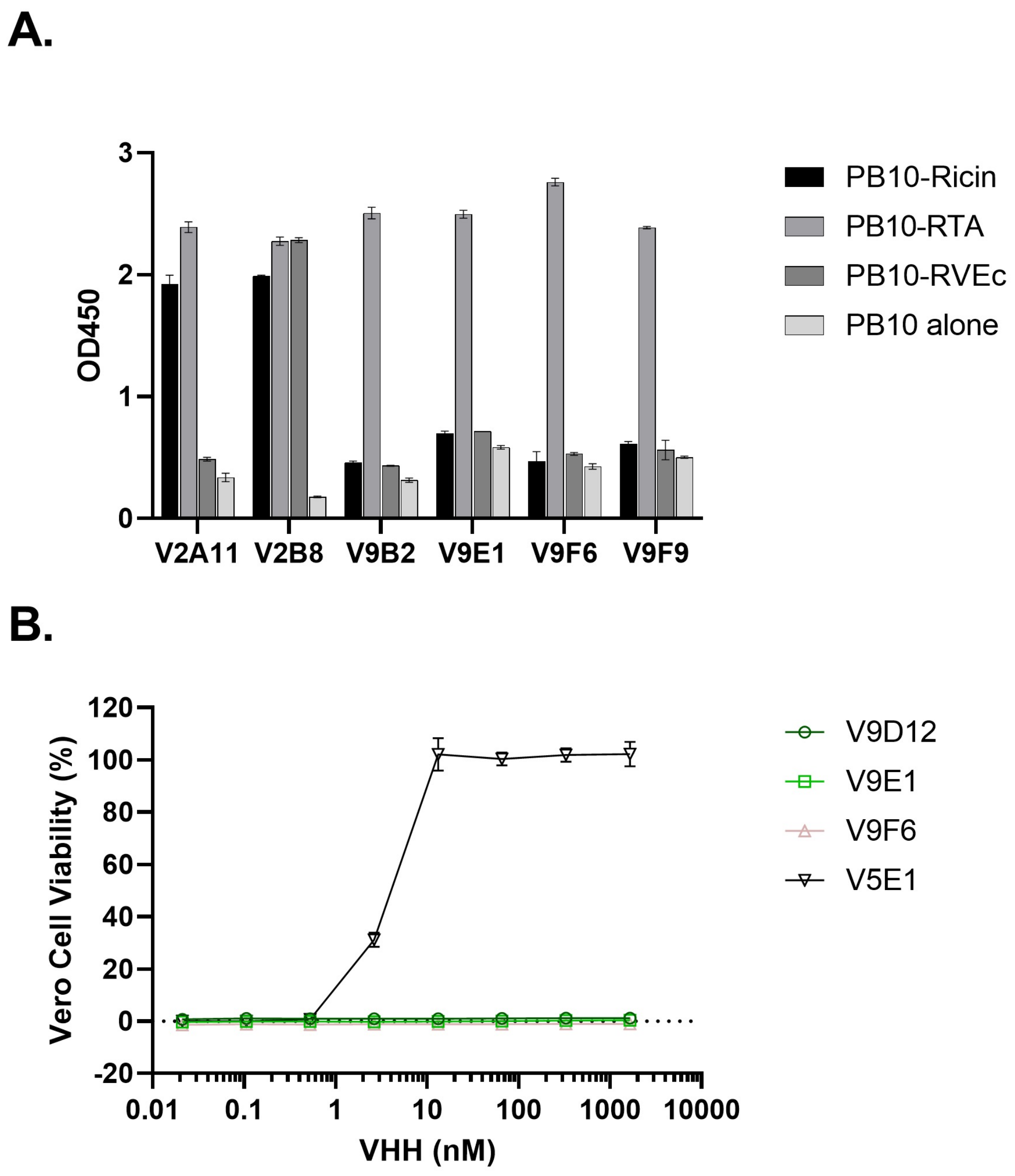

Figure S2. The V9 V$_H$Hs recognize the interface of RTA normally occluded by RTB. A) ELISA wells coated with mAb PB10 were overlaid with ricin, RTA, or RVEc and then probed with indicated VHHs. Control VHHs (V2A11, V2B8, V2B8) bound ricin holotoxin and RVEc, whereas the V9 VHHs only recognized RTA. B) Vero cell cytotoxicity assays demonstrateing that V5E1 but not the V9 VHHs are able to neutralize ricin toxin when mixed extracellularly.

FIGURE S3

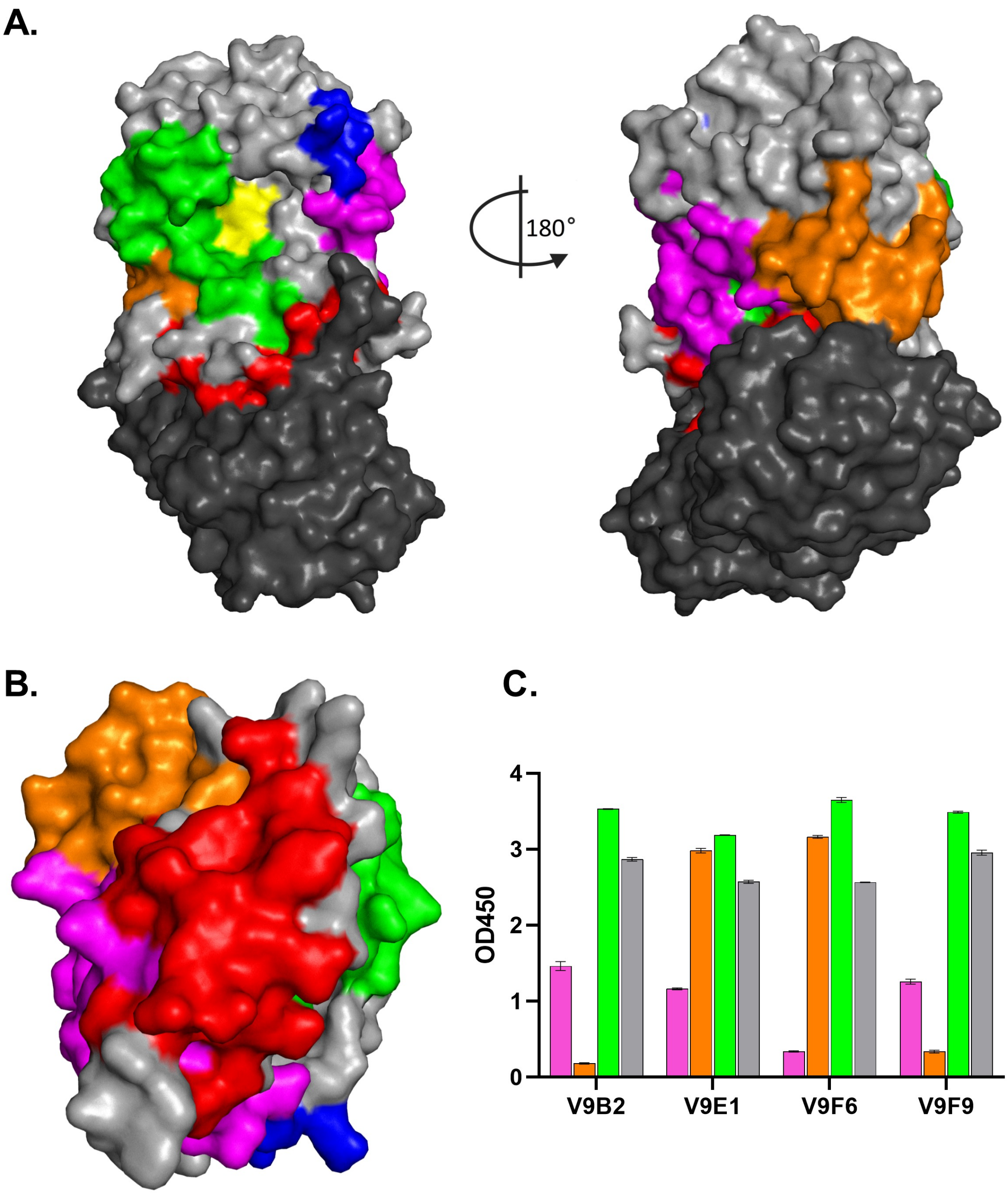

Figure S3. Anti-RTA mAb epitopes surround RTA's ribosome binding interface. A) Molecular surface of ricin, with RTA in light gray, RTB in dark gray, RTA's active site in yellow, and the epitopes of mAbs PB10 (blue) IB2 (green) SyH7 (orange) and JD4 (pink) colored. The residues on RTA that are covered by RTB (in whole or in part) are in red. The epitopes of SyH7, IB2 and JD4 surround the RTA-RTB interface and were used as landmarks to locate the relative binding locations of the V9 VHHs. B) RTA with RTB removed and viewed from RTB's binding location. The epitopes of SyH7 (orange, top left) JD4 (pink, bottom left) and IB2 (green, right) can be seen surrounding the residues that RTB covers (red). C) ELISA plates were coated with mAbs JD4 (pink bars), SyH7 (orange bars), IB2 (green bars) or WECB2 (control, gray bars), and then overlaid with RTA. VHHs were added, and binding (signifying lack of competition) was then detected with an anti-E-tag secondary antibody.

FIGURE S4

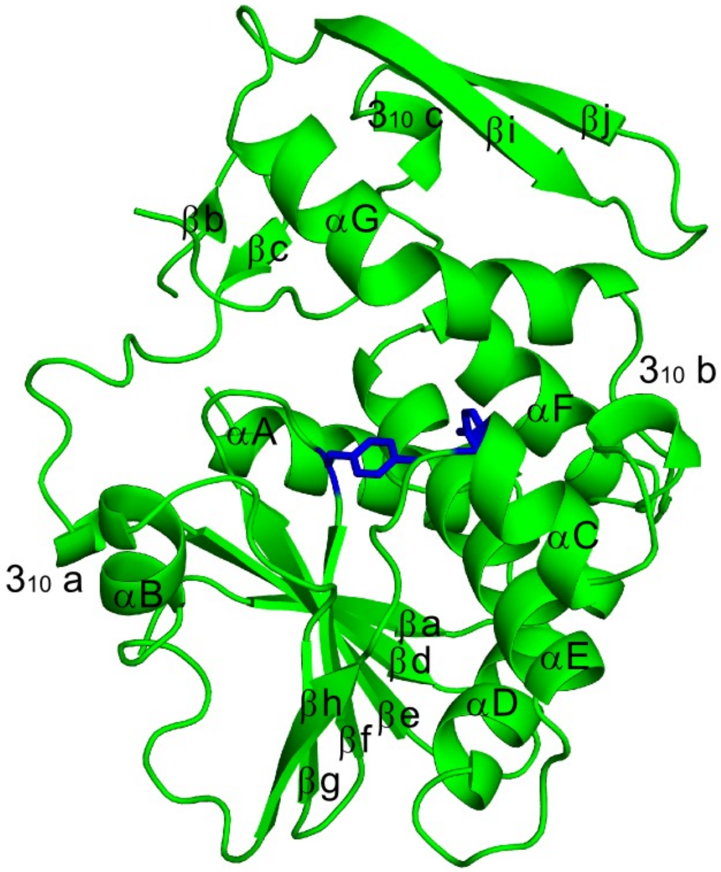

Figure S4. RTA secondary structural elements. Ribbon diagram of RTA in green with active site residues Tyr-80 and Tyr-123 drawn as all bonds colored blue (PDB 2AAI) with all secondary structures labeled.

FIGURE S5

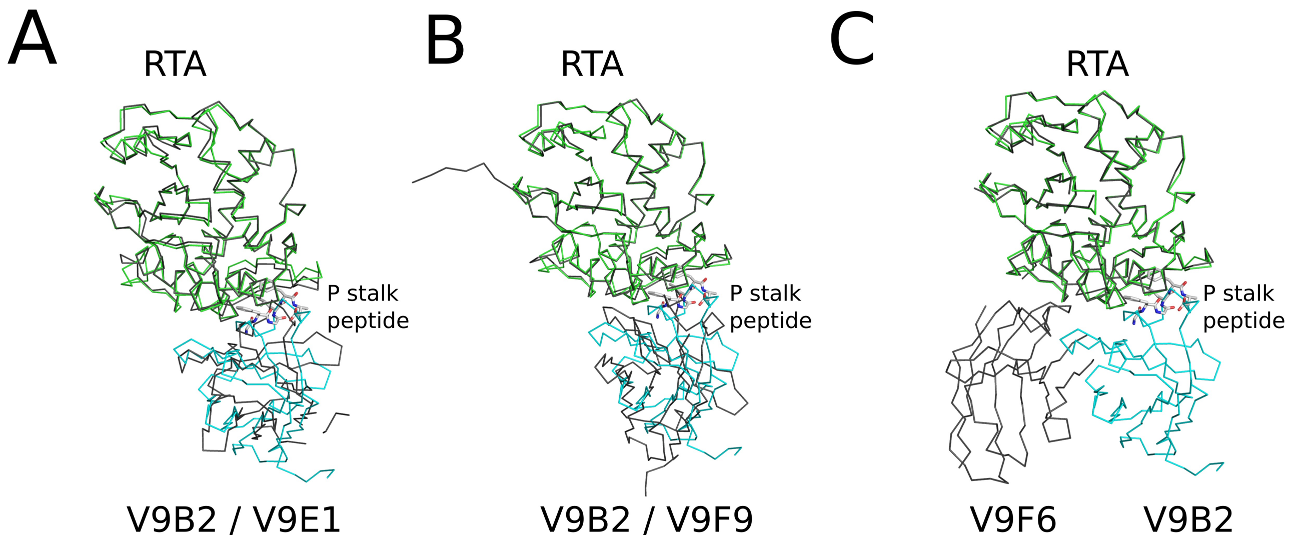

Figure S5. Comparison of V_HH interactions with P stalk binding site on RTA. (A) A) The superpositioned Ca-traces of (A) the RTA-V9B2 complex (green-cyan, respectively) with the RTA-V9E1 complex (gray-gray, respectively) and (B) the RTA-V9B2 complex (green-cyan, respectively) superposed onto the RTA-V9F9 complex (gray-gray, respectively) demonstrating the structural similarities and comparable contacts with the P stalk binding site of these two V_HHs. (C) The superpositioned Ca-traces of the RTA-V9B2 complex (green-cyan, respectively) with the RTA-V9F6 complex (gray-gray, respectively) illustrating the distinct contacts each V_HH has with RTA. The P stalk peptide is drawn as gray sticks with carbon atoms gray, nitrogen atoms blue, and oxygen atoms red.

FIGURE S6

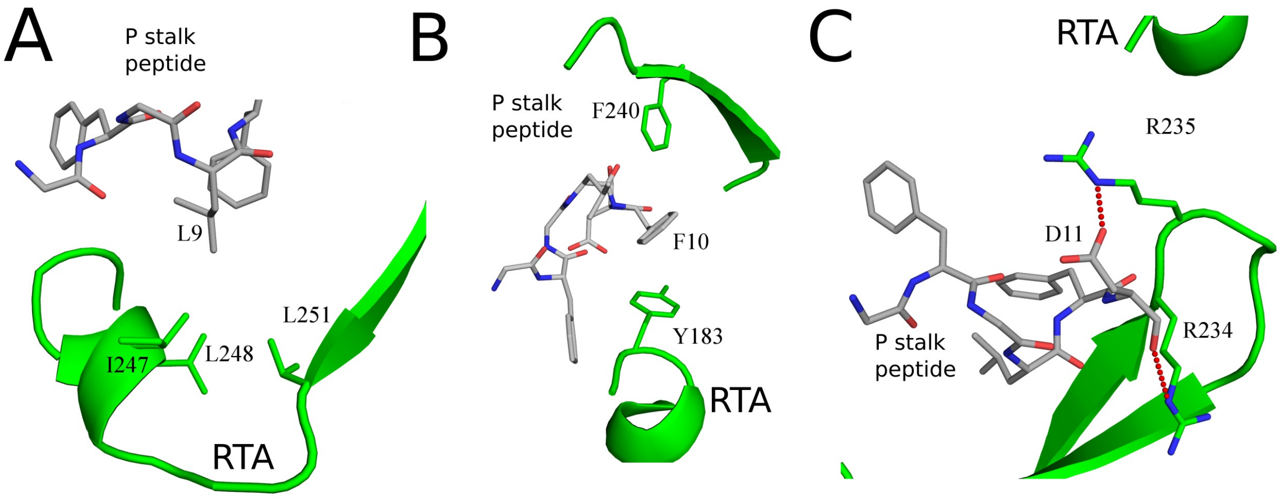

Figure S6. Key RTA-P stalk interactions. Ribbon diagram of RTA (green) showing key interactions of the P stalk binding site in RTA establishing (A) hydrophobic contacts to Leu-9 from the P stalk peptide, (B) p-stacking interactions with the P stalk residue Phe-10 and, (C) salt-bridges to the C-terminal Asp residue in the P stalk peptide.

FIGURE S7

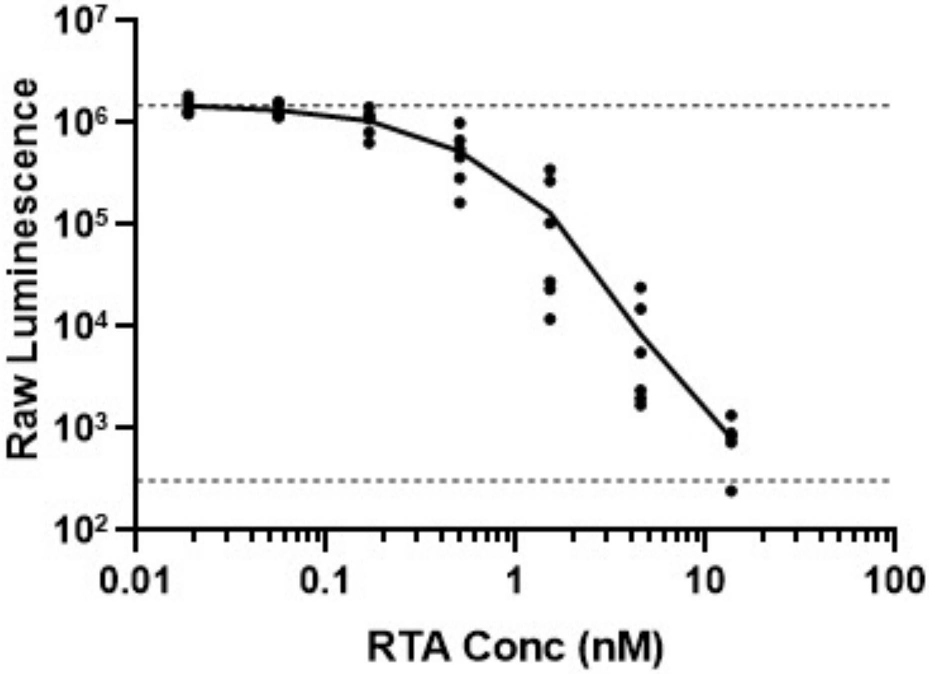

Figure S7. Dynamic range of *in vitro* translation assay across RTA doses. RTA was serially diluted from 13.6 to 0.019 nM and added to the IVT mixture with luciferase mRNA. Shown are the raw luminescence values determined by the SpectraMax iD3. Dashed lines indicate the average maximum and minimum luminescence values from positive control samples without RTA added and negative controls without luciferase mRNA added, respectively.

FIGURE S8

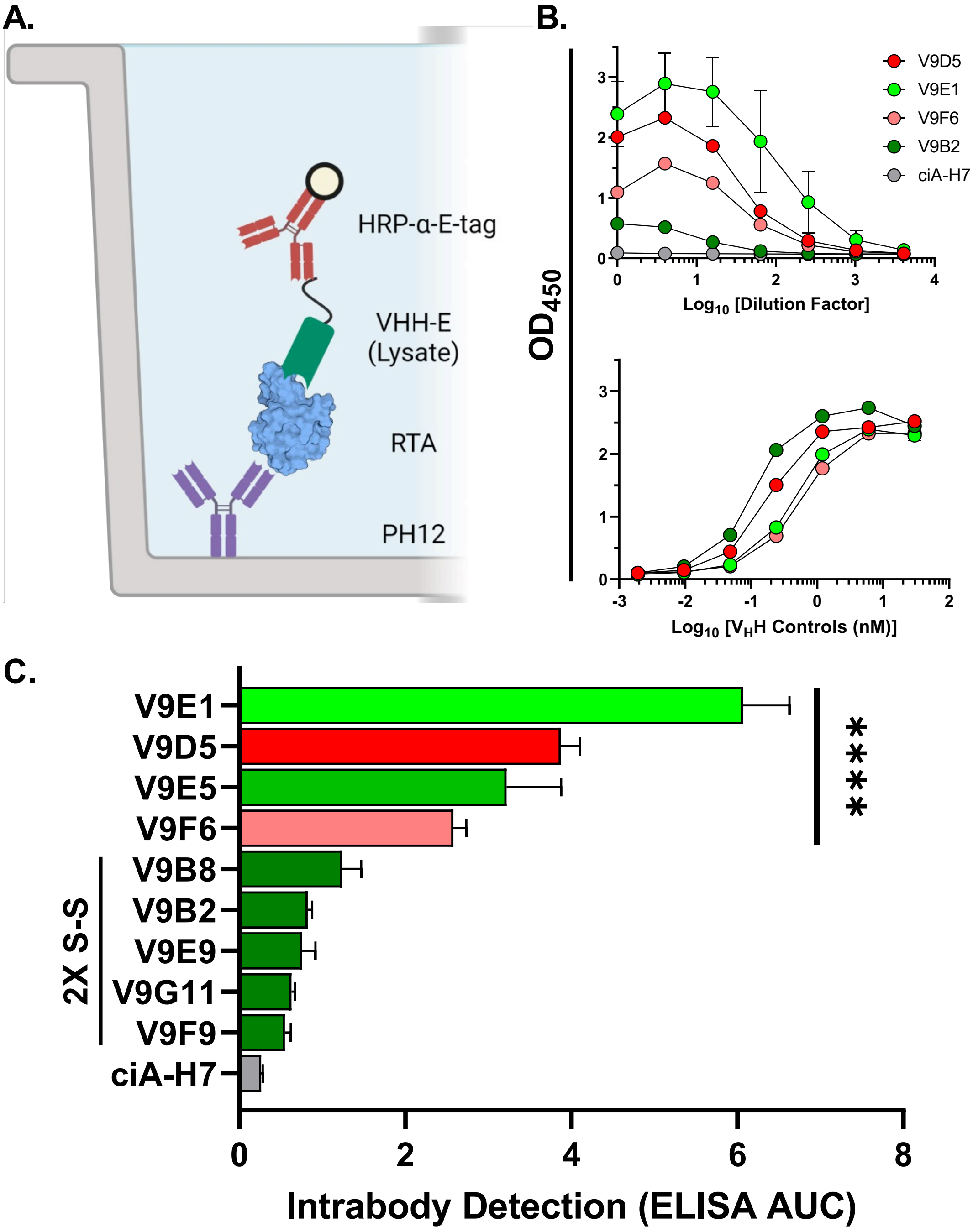

Figure S8. Intracellular expression of VHH intrabodies. (A) Intrabody ELISA detection schematic. (B, top) Vero cells were seeded in 6-well plates for one day then transiently transfected with V_HH-encoding pcDNA3.1 plasmids. Two days later, 4-fold serial dilutions of transfected cell lysates were applied to RTA-captured microtiter plates. Intracellular V_HH was detected using HRP-conjugated anti-E-tag antibody bound to C-terminal E-tags encoded on pcDNA3 expression vector. (B, bottom) Purified V_HH controls were 4-fold serially diluted, applied to RTA-captured microtiter plates and detected using HRP-conjugated anti-E-tag antibody. (C) AUC analysis of the Log_{10} transformed ELISA for all V9 intrabody-transfected cells.VHHs in dark green all contain two disulfide bonds. Significance was calculated using a one-way ANOVA with comparisons to ciA-H7 (negative control) shown. **** p<0.0001; *** p<0.001; ** p<0.01; * p<0.05.

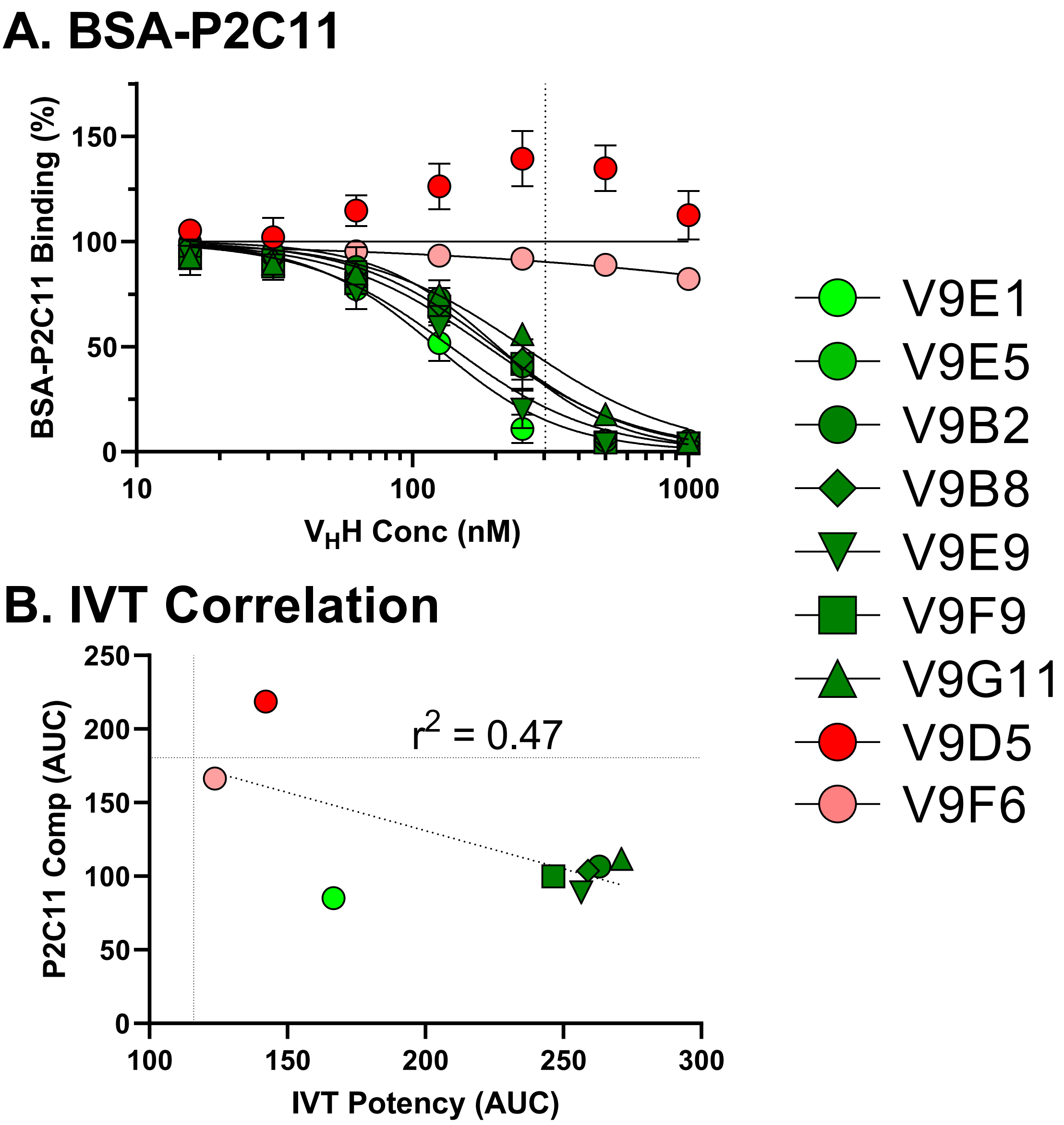

Figure S9. V9 V_HHs, except clan 5, prevent RTA-stalk binding. (A) V9 V_HHs were tested via ELISA for competition with BSA-P2C11 coated on microtiter plates, as described previously. OD_{450} values were normalized to wells in which RTA was added without competitor V_HH (100% binding). Vertical dashed line at 303 nM indicates the 1:1 molar ratio of competitor V_HH:RTA. Shown are the mean ± SD of at least three biological replicates. (B) AUC was determined for all V_HH competition profiles from (A) and a simple linear regression model was applied with IVT AUC results with r^2 values representing goodness of fit. Vertical and horizontal dotted lines represent the IVT standard curve AUC and 100% RTA binding (no competitor), respectively.

FIGURE S11

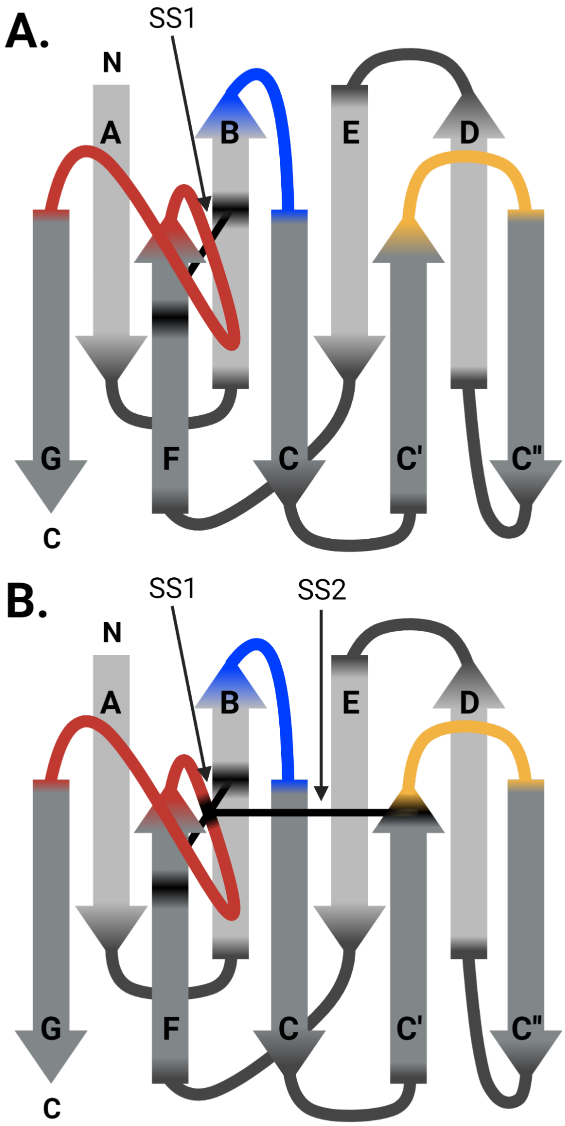

Figure S10. Topological comparison of high affinity vs. ultra-high affinity V9 VHHs. (A) High affinity V9 V_HHs in clans 1, 2, and 5 contain two cysteine residues in the framework regions upstream of CDR1 (blue) and CDR 3 (red) within β-strands B and F that can form an internal disulfide bond universally conserved among V_HHs ("SS1"; black line). (B) All ultra-high affinity V9 VHHs (clans 3 and 4) contain a surface-exposed disulfide bond ("SS2"; black line) between CDR3 (red) and the framework region immediately upstream of CDR2 (yellow) in addition to the internal disulfide bond, SS1. β-strands are labeled in accordance with Muyldermans 2013.

FIGURE S12

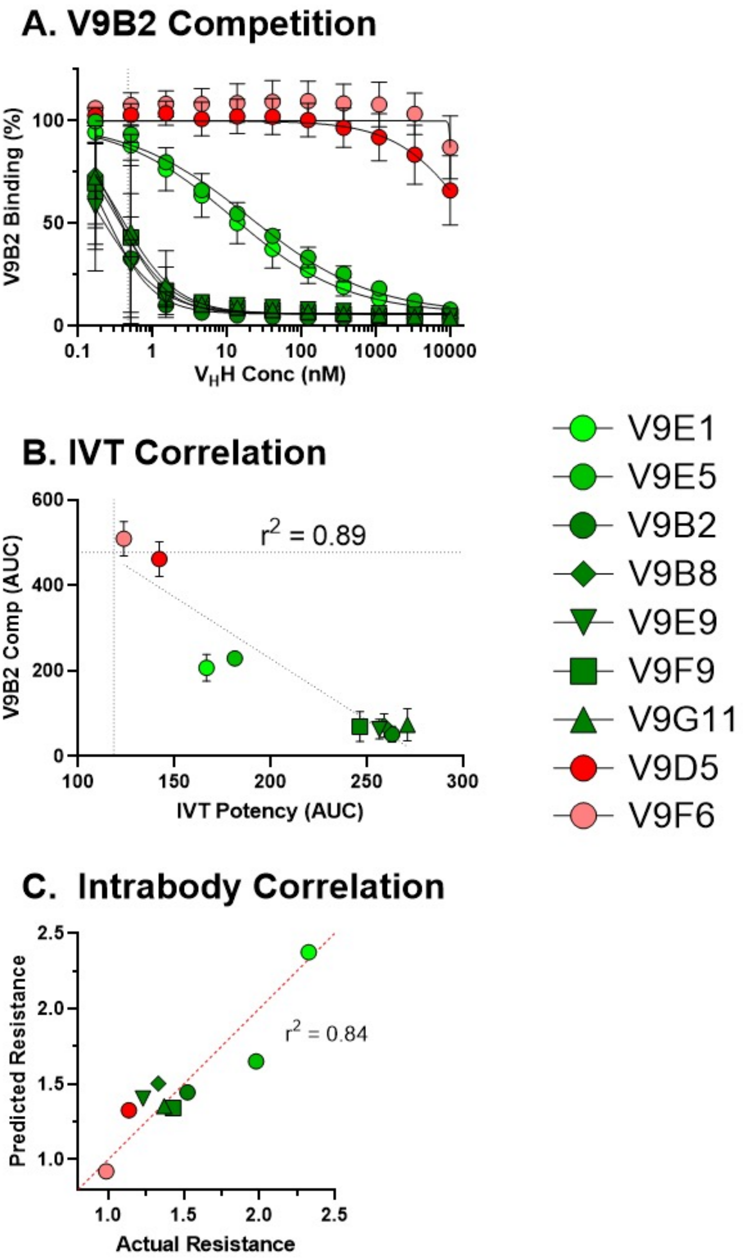

Figure S11. Binding site and strength correlates with RTA neutralization. (A) V9 V_HHs were tested via ELISA for competition with V9B2 (ultra-high affinity) coated on microtiter plates, as described previously. OD_{450} values were normalized to wells in which RTA was added without competitor V_HH (100% binding). Vertical dashed line at 0.46 nM indicates the 1:1 molar ratio of competitor V_HH:RTA. Shown are the mean ± SD of at least three biological replicates. (B) AUC was determined for all V_HH competition profiles from (A) and a simple linear regression model was applied with IVT AUC results with r^2 values representing goodness of fit. Vertical and horizontal dotted lines represent the IVT standard curve AUC and 100% RTA binding (no competitor), respectively. (C) Multiple linear regression analysis was performed to predict the resistance to ricin by cells transfected with V9 intrabodies based on their intracellular expression (Intrabody ELISA AUC) and binding profiles (V9B2 Competition AUC). Ricin resistance was defined by relative AUC values obtained from cytotoxicity experiments with 1 set to the AUC for control Vero cells treated with ricin.
